# A glycan-based approach to cell characterization and isolation: Hematopoiesis as a paradigm

**DOI:** 10.1084/jem.20212552

**Published:** 2022-09-06

**Authors:** Richard T. Piszczatowski, Emily Schwenger, Sriram Sundaravel, Catarina M. Stein, Yang Liu, Pamela Stanley, Amit Verma, Deyou Zheng, Ronald D. Seidel, Steven C. Almo, Robert A. Townley, Hannes E. Bülow, Ulrich Steidl

**Affiliations:** 1 Department of Cell Biology, Albert Einstein College of Medicine, Bronx, NY; 2 Department of Genetics, Albert Einstein College of Medicine, Bronx, NY; 3 Department of Biochemistry, Albert Einstein College of Medicine, Bronx, NY; 4 Department of Developmental and Molecular Biology, Albert Einstein College of Medicine, Bronx, NY; 5 Dominick P. Purpura Department of Neuroscience, Albert Einstein College of Medicine, Bronx, NY; 6 Departments of Oncology and Medicine, Albert Einstein College of Medicine-Montefiore Health System, Bronx, NY; 7 Montefiore Einstein Cancer Center, Albert Einstein College of Medicine-Montefiore Health System, Bronx, NY; 8 Department of Biological Sciences, University of Wisconsin Milwaukee, Milwaukee, WI; 9 Blood Cancer Institute, Albert Einstein College of Medicine, Bronx, NY; 10 The Saul R. Korey Department of Neurology, Albert Einstein College of Medicine, Bronx, NY; 11 Ruth L. and David S. Gottesman Institute for Stem Cell Research and Regenerative Medicine, Albert Einstein College of Medicine, Bronx, NY

## Abstract

Cell surfaces display a wide array of molecules that confer identity. While flow cytometry and cluster of differentiation (CD) markers have revolutionized cell characterization and purification, functionally heterogeneous cellular subtypes remain unresolvable by the CD marker system alone. Using hematopoietic lineages as a paradigm, we leverage the extraordinary molecular diversity of heparan sulfate (HS) glycans to establish cellular “glycotypes” by utilizing a panel of anti-HS single-chain variable fragment antibodies (scFvs). Prospective sorting with anti-HS scFvs identifies functionally distinct glycotypes within heterogeneous pools of mouse and human hematopoietic progenitor cells and enables further stratification of immunophenotypically pure megakaryocyte–erythrocyte progenitors. This stratification correlates with expression of a heptad of HS-related genes that is reflective of the HS epitope recognized by specific anti-HS scFvs. While we show that HS glycotyping provides an orthogonal set of tools for resolution of hematopoietic lineages, we anticipate broad utility of this approach in defining and isolating novel, viable cell types across diverse tissues and species.

## Teaser

Heparan sulfate (HS) glycotyping establishes a role for HS modification patterns in hematopoietic lineage differentiation in mouse and human, and provides an orthogonal approach to define and isolate viable cell types across different lineages and species.

## Introduction

Integral membrane proteins have long been used to characterize, classify, and select the cells on which they reside. Specifically, antibodies against cluster of differentiation (CD) markers utilized in the context of flow cytometry have revolutionized the definition and purification of distinct cell populations across various tissues. The hematopoietic system is a particularly diverse tissue comprised of a relatively well-understood hierarchy of stem, progenitor, and multiple mature cell lineages of varying functions. CD marker antibodies recognizing cell surface proteins, as well as a limited number of glycoconjugates have efficiently identified and isolated hematopoietic cell populations with unique functional properties (Akashi et al., 2000; Zhang et al., 2003; Oguro et al., 2013; Gabius et al., 2015). Notwithstanding, recent single-cell sequencing studies have revealed heterogeneity within many populations of cells defined as uniform by CD markers, including those within the hematopoietic lineage (Olsson et al., 2016; Lindahl et al., 2017; Karamitros et al., 2018; Chen et al., 2019; Wheat et al., 2020; Schwenger and Steidl, 2021). However, nucleic acid–based approaches are inherently destructive, rendering them unable to isolate, propagate, and analyze or functionalize live cells despite their resolving power. Consequently, novel approaches that distinguish and isolate viable cells from heterogeneous pools at a high resolution are urgently needed.

**Figure 1.**
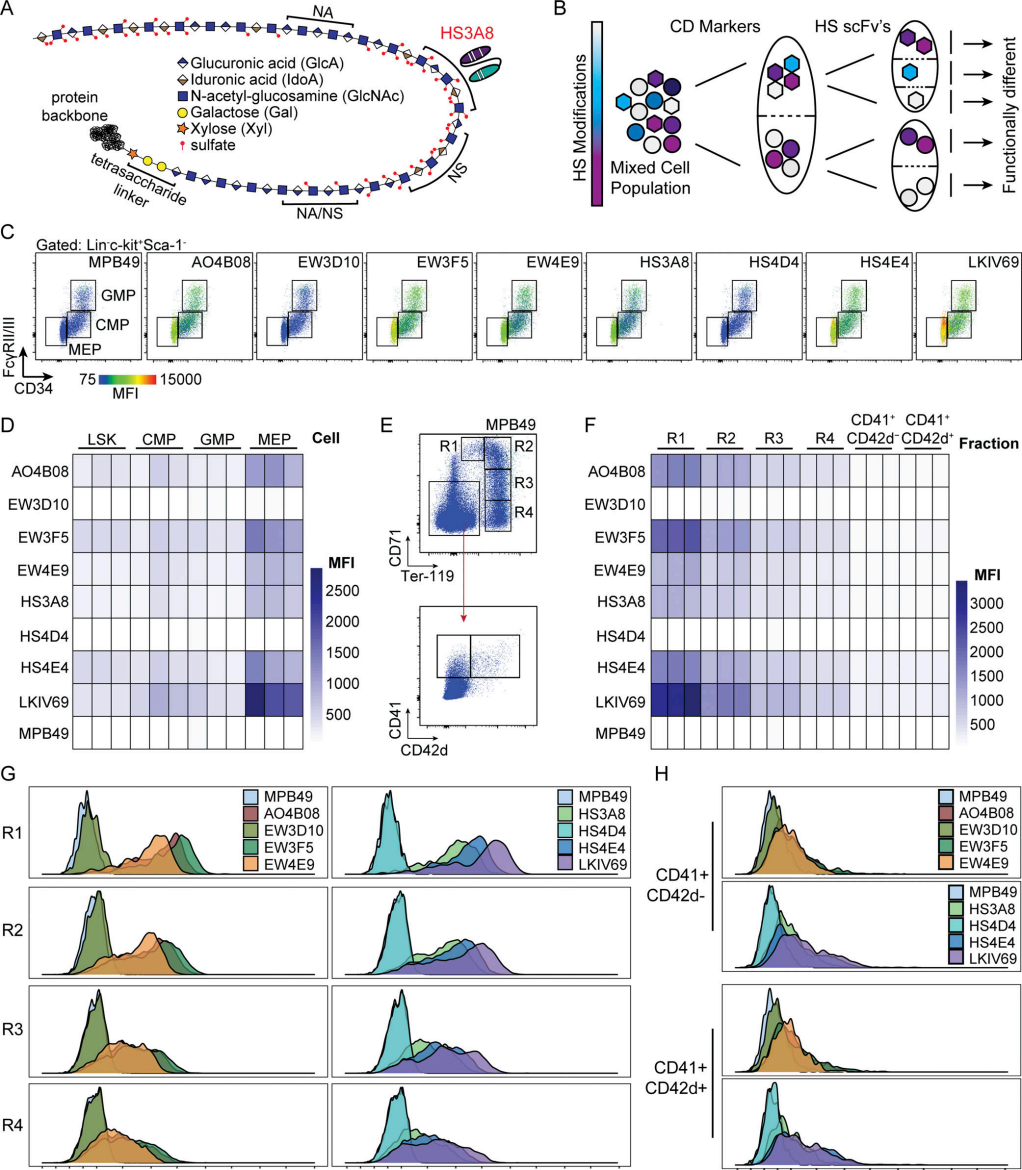
**A panel of HS-specific scFv antibodies defines distinct glycotypes of hematopoietic cells and reveals divergent HS glycotypes between megakaryocyte and erythroid lineages. (A)** Schematic of an HS polysaccharide chain attached to a protein backbone. The repeating disaccharide of hexuronic acid and glucosamine, as well as the characteristic tetra-saccharide linker region, are indicated. Red circles denote positions of sulfations that establish *N*-acetylated (NA) domains, *N*-acetylated/*N*-sulfated (NA/NS) domains, and completely *N*-sulfated (NS) domains. Depicted is a proposed binding position for the HS3A8 HS scFv along the HS glycan chain. **(B)** Conceptual diagram of HS-specific scFvs as an orthogonal tool to the existing CD marker separation approach. **(C)** Representative flow cytometry plots of each HS scFv within hematopoietic progenitor populations resolved by canonical CD marker gating into CMPs (Lin^-^ckit^+^CD34^+^FcγRII/III^lo^), GMPs (Lin^−^ckit^+^CD34^+^FcγRII/III^hi^), and MEPs (Lin^−^ckit^+^CD34^−^FcγRII/III^lo^) (*n* = 3). The color coding of scFv-binding MFI is shown at the bottom left. The MPB49 scFv has no known epitope and serves as a negative control. **(D)** Heatmap of the MFI of HS scFv binding within LSK (Lin^−^ckit^+^Sca-1^+^), CMP, GMP, and MEP populations. Each column represents one biological replicate (*n* = 3). **(E)** CD marker gating scheme and MPB49 signal of total mouse bone marrow resolved into terminally differentiating erythroid fractions R1–R4 (by CD71 and Ter-119; Socolovsky et al., 2001; Zhang et al., 2003), and terminally maturing megakaryocyte fractions (by CD41 and CD42d; Psaila et al., 2016). **(F)** Heatmap of the MFI of HS scFv binding within R1–R4 erythroid fractions and terminally differentiating megakaryocyte fractions. Each column represents one biological replicate (*n* = 3). **(G)** Distribution of the binding of each HS-specific scFv within the R1–R4 erythroid fractions and within terminally differentiating megakaryocytes. **(H)** Distribution of the binding of each HS-specific scFv within the CD41^+^CD42d^−^ and CD41^+^CD42d^+^ megakaryocyte fractions.

**Figure S1.**
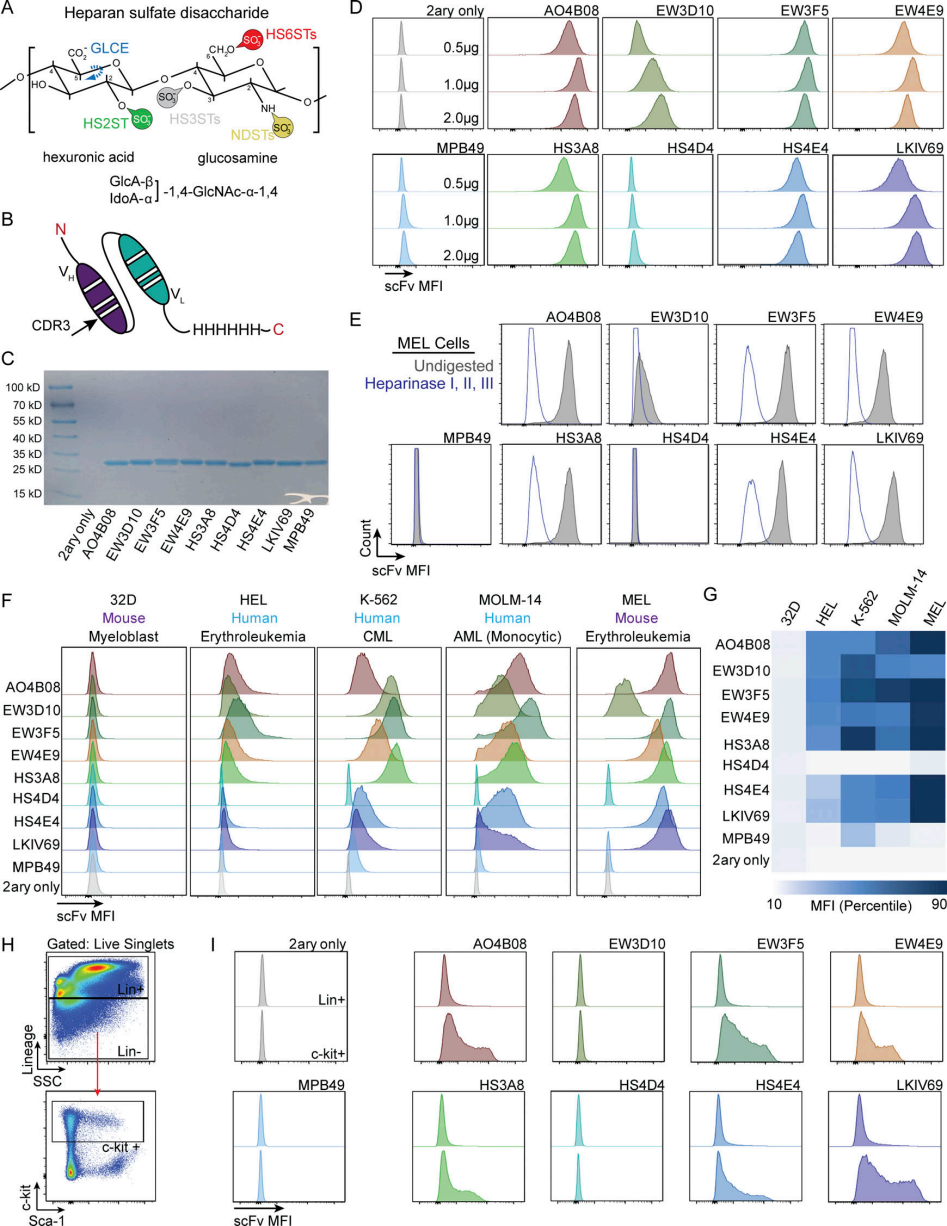
**Production and optimization of flow cytometry compatible HS scFvs and distinct binding signatures of murine and human hematopoietic cell lines and murine bone marrow. (A)** Characteristic HS disaccharide comprising a hexuronic acid and a glucosamine. Indicated are positions of the sugar moieties that can be modified by specific HS modification enzymes, including GLCE (C5-glucuronyl-epimerase), HS2ST (HS-2-*O*-sulfotransferase), HS3STs (HS-3-*O*-sulfotransferases, encoded by seven genes in vertebrates: Hs3st1,2,3A,3B,4,5,6), HS6STs (HS-6-*O*-sulfotransferases, encoded by three genes in vertebrates: Hs6st1,2,3), and NDSTs (*N*-deacetylase-*N*-sulfotransferase, encoded by four genes in vertebrates: Ndst1,2,3,4). **(B)** Schematic of a His-tagged HS scFv, with heavy (V_H_) and light (V_L_) chains depicted. The CDR3 sequence indicated provides specificity for each scFv. **(C)** Coomassie gel of 1 μg of each HS scFv after purification. **(D)** Representative histogram plots showing titration of HS scFvs using the MEL cell line at 0.5, 1.0, and 2.0 μg per reaction (*n* = 3). **(E)** Representative histogram plots of HS scFv binding in MEL cells with or without pre-digestion with a Heparinase I, II, III cocktail (undigested in gray, digested in blue; *n* = 3). **(F)** Representative histograms of the binding of each HS scFv across multiple hematopoietic cell lines (*n* = 3). The MPB49 scFv has no known epitope and serves as a negative control. **(G)** Heatmap of the average MFI percentile for each scFv across multiple hematopoietic cell lines (*n* = 3). **(H)** Gating schematic for the analysis of HS scFv binding within mature (Lin^+^) and HSPC (Lin^−^c-kit^+^) populations within mouse bone marrow. **(I)** Representative histograms of HS scFv binding within Lin^+^ and HSPC populations of mouse bone marrow.

Heparan sulfate proteoglycans (HSPGs) are proteins with covalently linked heparan sulfate (HS) glycan chains that are present in all living animals and serve a multitude of functions in development and physiology (Bülow and Hobert, 2006; Bishop et al., 2007; Sarrazin et al., 2011). HS are linear glycans of repeating hexuronic-*N*-acetylglucosamine disaccharides with highly complex modification patterns resulting from near limitless combinatorial possibilities of modifications such as sulfation, deacetylation, and epimerization of glycan moieties along the chains (Turnbull et al., 2001; Esko and Selleck, 2002; Lindahl and Li, 2009; Fig. 1 A and Fig. S1 A). Importantly, some proteoglycans such as CD44 exist in heparan sulfate–specific isoforms (Bennett et al., 1995; Jackson et al., 1995), which are indistinguishable when using conventional CD44 FACS antibodies. Therefore, our proposed glycotyping approach has the potential to reveal a dimension of cell characterization not achievable by CD markers alone. Genetic, biochemical, and structural studies show that specific HS modification patterns regulate cell–cell signaling, for example, by modulation of receptor–ligand binding (Lindahl and Li, 2009; Xu and Esko, 2014; Townley and Bülow, 2018). Collectively, these findings have led to the concept of a heparan sulfate code, in which cellular HS modification patterns help define cellular identity and interactions (Habuchi et al., 2004; Bülow and Hobert, 2006; Lamanna et al., 2007). To analyze cellular distribution patterns of HS, antibody stains were performed. Due to the inherent weak non-immunogenicity of HS, the antibodies used in these experiments were anti-HS single-chain variable fragment antibodies (scFvs) isolated by panning from phage display libraries (Dennissen et al., 2002; van de Westerlo et al., 2002; van Kuppevelt et al., 1998). These scFvs, which recognize different combinations of HS modifications, revealed surprising cellular specificity of HS patterns across species, supporting the idea of cell-specific HS distribution patterns, which in nematodes could be even single-cell specific (van Kuppevelt et al., 1998; Dennissen et al., 2002; van de Westerlo et al., 2002; Attreed et al., 2012). In addition, flow cytometry experiments using these scFvs identified transient HS epitopes in differentiating mouse embryonic stem cells in culture (Lamanna et al., 2006; Baldwin et al., 2008).

HS have also been detected in hematopoietic cell lines (Stöcker et al., 1996; Drzeniek et al., 1999), and hematopoietic cytokines including PF4, IL-8, G-CSF, among others, have demonstrated HS-binding activity (Stringer and Gallagher, 1997; Spillmann et al., 1998; Wettreich et al., 1999; Frevert et al., 2003; Sebollela et al., 2005; Murphy et al., 2007; Lord et al., 2017). Moreover, HS are important for homeostasis of the hematopoietic system, including at the stem and progenitor level (Netelenbos et al., 2002; Holley et al., 2011; Saez et al., 2014). Here, we use a library of HS-specific, flow cytometry–compatible scFvs to establish binding patterns, herein referred to as glycotypes, of cell populations within the hematopoietic system. Our glycotyping strategy, in combination with the CD marker system, identified new hematopoietic cell populations in the megakaryocyte and erythroid lineages of both mice and humans. These cell populations were functionally and transcriptionally distinct and could be isolated from both immunophenotypically homogeneous as well as heterogeneous progenitor cell populations in a non-destructive manner. Overall, HS glycotyping provides an orthogonal approach for the isolation of unique cell types.

## Results

### Murine hematopoietic lineages display unique HS glycotypes

To systematically leverage the molecular diversity of HS for cell identification and characterization, we focused on a panel of eight scFvs previously shown to recognize different combinations and arrangements of HS modifications, i.e., HS modification patterns or epitopes (Table 1; Dennissen et al., 2002; van de Westerlo et al., 2002). These studies also showed that different HS-specific scFvs displayed distinct staining patterns in tissue sections suggesting a high level of cellular specificity. We therefore hypothesized that by using the HS-specific scFvs, it may be possible to define a given cell population by the sum of the qualitative and quantitative binding of a panel of scFvs, thereby establishing cellular “glycotypes.” We also envisioned that applying a set of HS-specific scFvs (Fig. S1, A and B; and Fig. 1 A) in combination with the CD marker system could offer a conceptually orthogonal avenue for the phenotypic and functional separation of heterogeneous populations of cells (Fig. 1 B). In proof-of-concept experiments, we first engineered a 6× histidine tag at the C-terminus of each scFv and tested them for glycotyping hematopoietic cell lines using FACS, similar to experiments conducted with mouse embryonic stem cells (Fig. S1, B–D; Lamanna et al., 2006; Baldwin et al., 2008). We found that binding of the scFvs to cells was HS-dependent as neither cells exposed to enzymatic digestion with heparinases nor 32D cells (reported to lack HSPGs [Roghani et al., 1994; Richard et al., 1995; Rubin et al., 2001]) exhibited scFv binding (Fig. S1, E and F). Intriguingly, the panel of eight HS-specific scFvs, but not a control scFv (MPB49), reproducibly exhibited unique qualitative and quantitative binding patterns across a set of common mouse and human hematopoietic cell lines (Fig. S1, F and G; and Table S1) demonstrating the discriminatory power of the glycotyping approach in principle.

**Table 1.** HS binding scFv antibodies and their characteristics

scFv name	V_H_ family	CDR3 sequence	HS modifications required for binding^a^
AO4B08	DP-47	SLRMNGWRAHQ	*N*-Sulfation, *C5*-epimerization, 2-*O*-Sulfation, 6-*O*-Sulfation
EW3D10	DP-38	GRTVGRN	*N-Sulfation, 6-O*-sulfation
EW3F5	DP-38	SGRQARQGRFPK	Unknown
EW4E9	DP-38	LRGTKMFRH	Unknown
HS3A8	DP-38	GMRPRL	*N*-sulfation, *C5*-epimerization, 2-*O*-sulfation, 6-*O*-sulfation (likely)
HS4D4	DP-58	GMRPRL	*N*-sulfation/*N*-Acetylated, *C5*-epimerization, 2-*O*-sulfation (likely), 6-*O*-sulfation (likely)
HS4E4	DP-38	HAPLRNTRTNT	*N*-sulfation/*N*-Acetylated, *C5*-epimerization, 2-*O*-sulfation, 6-*O*-sulfation
LKIV69	DP-38	GSRSSR	*N*-Sulfation, *C5*-epimerization, 2-*O*-Sulfation
*MPB49* ^b^	DP-38	WRNDRQ	No known epitope (negative control)

Heparan sulfate modifications required for scFv binding. CDR3 is the complementarity determining region three within the variable heavy chain.

a Compiled from Attreed et al. (2016).

b MPB49 has no known epitope, does not appear to bind HS, and serves as a negative control.

**Figure S2.**
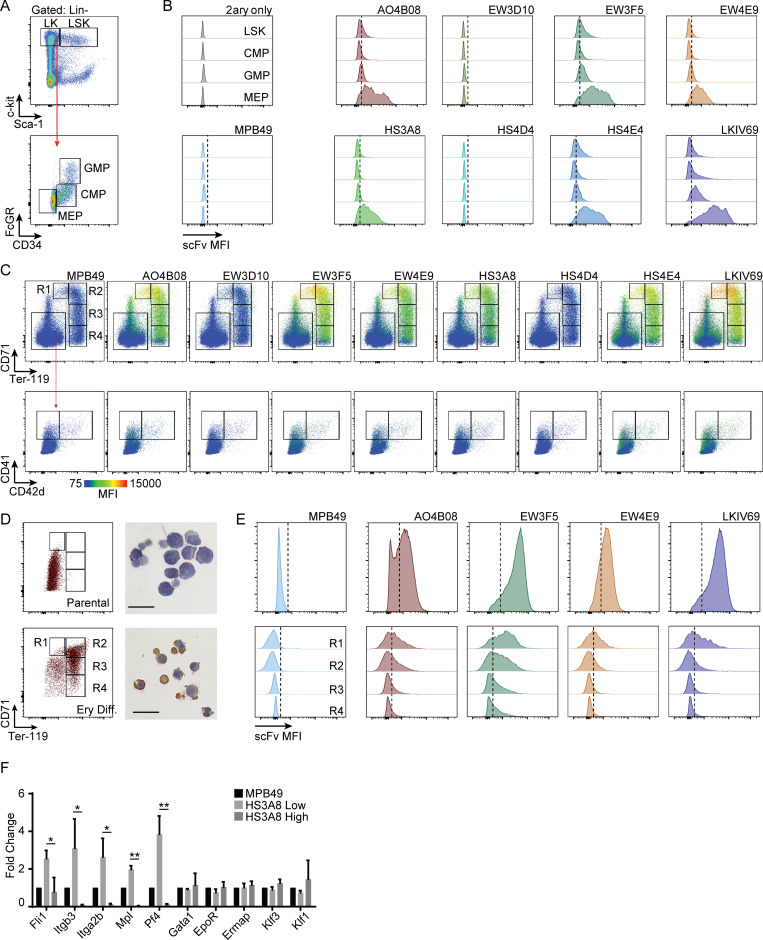
**HS glycotyping reveals distinct modification patterning in murine hematopoietic progenitor populations and within megakaryocyte and erythrocyte differentiation. (A)** Gating schematic for the analysis of HS modification expression within HSPC subpopulations **(B)** Representative histograms of HS scFv binding within LSK (Lin^−^c-kit^+^Sca-1^+^), CMP (Lin^−^ckit^+^CD34^+^FcγRII/III^lo^), GMP (Lin^−^ckit^+^CD34^+^FcγRII/III^hi^), and MEP (Lin^−^ckit^+^CD34^−^FcγRII/III^lo^) populations. **(C)** Representative flow cytometry plots of HS scFv signal overlaid onto FACS defined R1–R4 erythroid fractions (upper panel) and megakaryocytic populations (lower panel). The color code represents scFv-binding MFI  (n=3). Control panels for MPB49 are identical to the panels in Fig. 1 C and are shown for comparison only. **(D)** FACS profile and benzidine staining of parental (upper panel) and erythroid differentiated (lower panel) ES-EP cells. Erythroid differentiated ES-EP’s fall under the archetypal R1–R4 FACS gating scheme. Scale bar indicates 20 μm. **(E)** Representative histograms of HS scFv binding within parental ES-EP’s (upper panel) and the R1–R4 fractions derived from ES-EPs differentiated down the erythroid lineage (*n* = 3). **(F)** qRT-PCR analysis of canonical megakaryocyte and erythroid genes compared between HS3A8-high vs. -low sorted MEPs (*n* = 3–5 per gene). Error bars represent ± SD; *, P < 0.05; **, P < 0.01.

We next investigated the binding patterns of HS-specific scFvs in primary cells of the murine hematopoietic system separated by established CD markers (Table S2). We observed consistently higher HS scFv binding to hematopoietic stem and progenitor cell (HSPC; Lin^−^c-kit^+^) populations as compared to mature hematopoietic cells (Fig. S1, H and I). Within the pool of committed progenitor populations (Lin^−^c-kit^+^Sca-1^−^), we observed distinct glycotypes for each of the common myeloid progenitor (CMP), granulocyte–monocyte progenitor (GMP), and megakaryocyte–erythrocyte progenitor (MEP) populations (Fig. 1, C and D; and Fig. S2, A and B). We decided to focus on the megakaryocyte–erythroid lineage because, despite all advances with existing CD marker schemes, the associated progenitor populations remain comparatively heterogeneous. We discovered that terminally differentiating erythroid and megakaryocytic populations (as defined by canonical CD markers CD71, Ter-119, and CD41, CD42d, respectively [Socolovsky et al., 2001; Zhang et al., 2003; Psaila et al., 2016]), displayed a dichotomization of HS scFv binding. A subset of committed erythroid progenitors displayed high levels of binding for several HS scFvs, which diminished upon terminal differentiation, while megakaryocytes showed continuously minimal levels of binding (Fig. 1, E–H, and Fig. S2 C). Longitudinal modeling of terminal erythroid differentiation using a mouse embryonic stem cell–based erythroid progenitor differentiation cell line (ES-EP) system (Carotta et al., 2004; Choe et al., 2010) revealed similar dynamic HS scFv binding patterns (Fig. S2, D and E). Taken together, these data demonstrate that a panel of eight HS-specific scFvs provide binding signatures with sufficient discriminatory power to define glycotypes of mouse hematopoietic cell types and reveal dynamic expression of specific HS modification patterns during erythroid differentiation.

### HS modification patterns distinguish phenotypically and functionally distinct cell types within the MEP population

**Figure 2.**
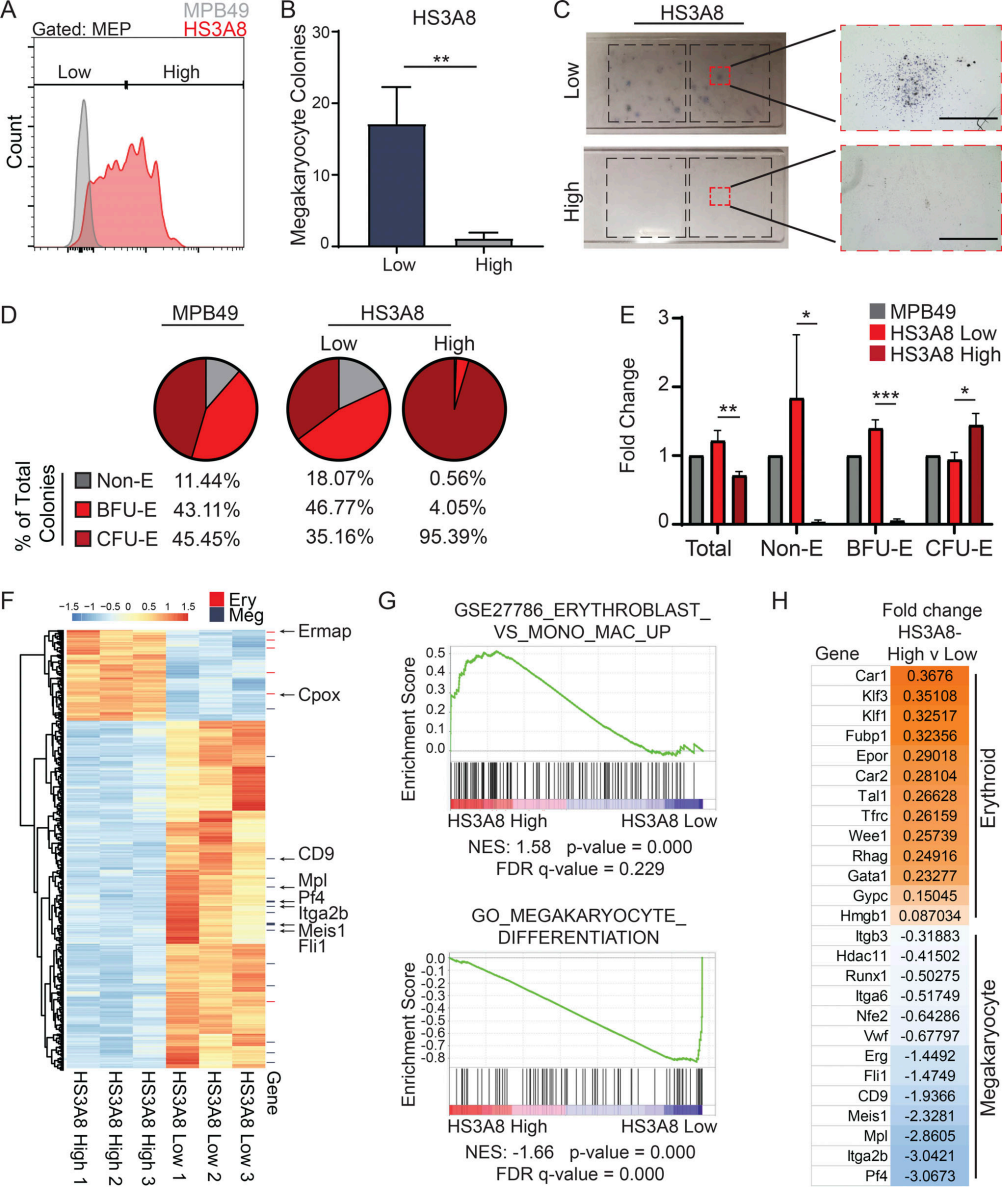
**Distinct functional phenotypes of MEPs separated by HS scFv binding. (A)** Sorting of MEPs (Lin^−^c-kit^+^CD34^−^FcγRII/III^lo^) into HS3A8-low binding (bottom 50%) and HS3A8-high binding (top 50%) populations. The gray histogram shows the MPB49 (non HS-binding scFv) control. **(B)** Megakaryocyte colony forming potential of sorted HS3A8-high/low MEPs (*n* = 3). **(C)** Representative MegaCult assay of HS3A8 sorted MEPs. Right-side panels show magnified images of acetylcholinesterase positive megakaryocyte colonies generated by sorted HS3A8-low MEPs (upper panels), and predominantly small erythroid colonies generated by sorted HS3A8-high MEPs (lower panels). Scale bars indicate 400 μm. **(D)** Average percentages of colony types formed by HS3A8-low vs. -high sorted MEPs (MPB49 control mock-sorted MEPs as a comparison) in functional erythroid colony forming assay (*n* = 3). **(E)** Fold change of erythroid colony formation within sorted MPB49 and HS3A8-high/low MEPs. **(F)** Color coded heatmap of the top 500 differentially regulated genes in sorted HS3A8-low vs. -high MEPs (*n* = 3 individual mice). Red and navy annotation lines indicate erythroid and megakaryocyte genes, respectively. **(G)** GSEA of differentially expressed genes in HS3A8 sorted MEPs identified positive enrichment of erythroid (upper) and megakaryocytic (lower) gene expression signatures in HS3A8-high and HS3A8-low sorted MEPs, respectively. **(H)** List of canonical erythroid and megakaryocytic genes and their respective fold change in expression between HS3A8-high and -low sorted MEPs. Bars represent mean values. Error bars represent ± SD; *, P < 0.05; **, P < 0.01; ***, P < 0.001.

We next tested whether HS scFvs allow separation of hematopoietic populations into functionally distinct subsets of cells. To this end, we focused on the HS3A8 scFv as it (1) had existing information available regarding binding specificity (see below), (2) bound MEPs to a varying degree with substantial dispersion, and (3) displayed dynamic binding within the spectrum of hematopoietic populations. We utilized multi-parameter high-speed cell sorting to separate immunophenotypic MEPs into fractions with either high (top 50%) or low (bottom 50%) binding of HS3A8 (Fig. 2 A). Strikingly, functional megakaryocyte colony-forming potential was found almost exclusively in HS3A8-low binding MEPs (Fig. 2, B and C). In contrast, the HS3A8-high binding fraction gave rise to predominantly (over 95% of total colonies) small and mature CFU-E (CFU-erythroid) colonies (Fig. 2, D and E). HS3A8-low binding cells formed immature burst forming units-erythroid (BFU-E) and mature (CFU-E) erythroid colonies in roughly equal numbers, similar to cells gated on the MPB49 (negative control) scFv. These data indicate that the HS modifications recognized by HS3A8 are associated with functionally distinct subsets of cells and show developmental divergence within MEPs based on expression of HS modification patterns.

To define the molecular features associated with HS3A8-sorted MEPs, we performed bulk RNA sequencing (RNA-seq) of FACS-sorted HS3A8-high and HS3A8-low MEPs. Hierarchical clustering of transcripts revealed segregation of a large cohort of differentially expressed genes between the two groups, including numerous canonical erythroid and megakaryocyte genes (Fig. 2 F). Moreover, gene set enrichment analysis (GSEA) identified characteristic erythroid and megakaryocytic gene signatures in the HS3A8-high and HS3A8-low binding cells within MEPs, respectively (Fig. 2 G). We further confirmed the expression of several bona fide megakaryocyte and erythroid marker genes in HS3A8-high vs. HS3A8-low sorted MEPs by quantitative RT-PCR (qRT-PCR; Fig. 2 H and Fig. S2 F). In sum, the HS glycotyping approach identified functionally and molecularly distinct subsets of megakaryocytic and erythroid cells within the MEP population as defined by conventional immunophenotypic means.

### HS scFv-defined HSPC subsets have functionally distinct properties in vivo

**Figure 3.**
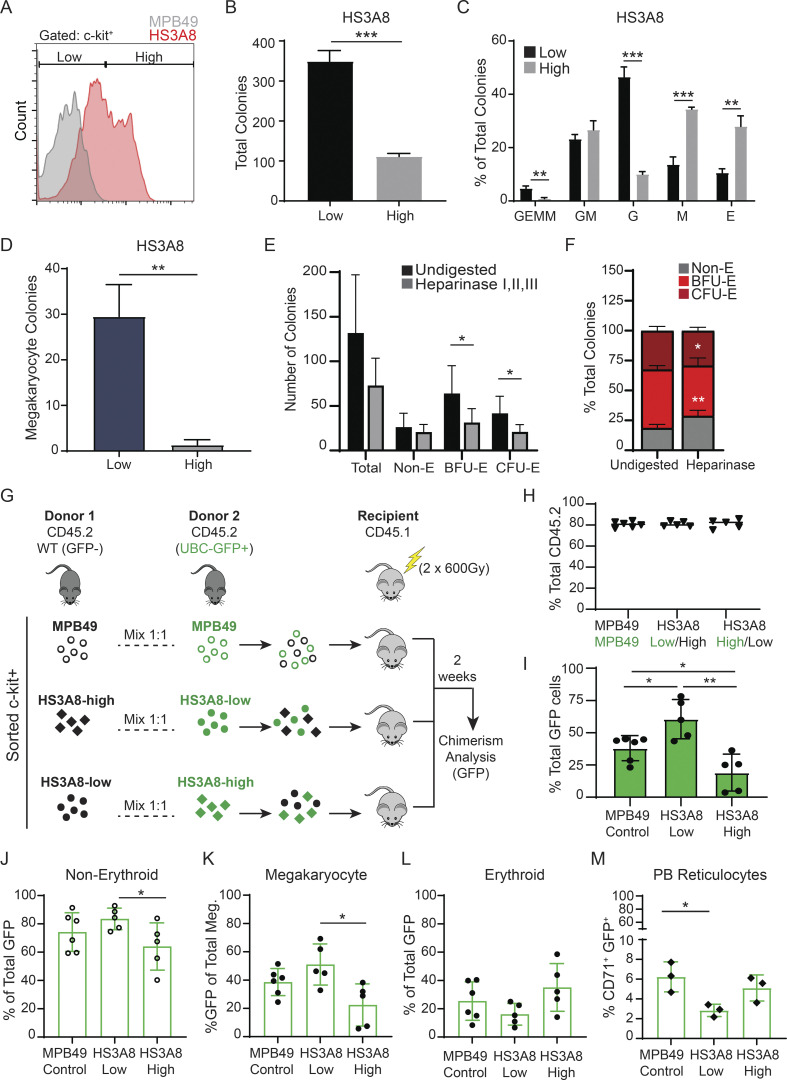
**Functional separation of a heterogeneous HSPC pool by HS scFv binding. (A)** Sorting of c-kit^+^ (Lin^−^c-kit^+^) HSPCs into low (bottom 50%) and high (top 50%) populations based on binding of HS3A8. The gray histogram shows the MPB49 (non HS-binding scFv) control. **(B)** Total colony forming capacity of sorted c-kit^+^ HS3A8-high vs. -low cells. **(C)** Percentage of colony types generated by c-kit^+^ HS3A8-high/low sorted HSPCs (*n* = 3). **(D)** Megakaryocyte colony forming potential of c-kit^+^ HS3A8-high/low sorted HSPCs (*n* = 3). GEMM, granulocyte, erythrocyte, monocyte, megakaryocyte CFU; GM, granulocyte, monocyte CFU; G, granulocyte CFU; M, monocyte CFU; E, erythrocyte CFU. **(E)** Erythroid colony formation of sorted c-kit^+^ (Lin^−^c-kit^+^) cells with or without digestion by a Heparinase I, II, III cocktail (*n* = 6). **(F)** Percentages of colony types from erythroid colony forming assay of sorted c-kit^+^ (Lin^−^c-kit^+^) cells with or without digestion by a Heparinase I, II, III cocktail (*n* = 6). **(G)** Outline of competitive transplantation of HS scFv-sorted HSPCs. MPB49 control stained and mock-sorted HSPCs from non-GFP and GFP donors (expressing GFP ubiquitously under control of the ubiquitin-C promoter [UBC-GFP^+^]) were equally mixed and transplanted into lethally irradiated congenic recipients. HS3A8-low sorted HSPCs from a UBC-GFP^+^ donor were equally mixed with HS3A8-high sorted HSPCs from a non-GFP donor (and vice versa) and then transplanted. **(H)** Total donor engraftment in bone marrow of transplanted recipients 2 wk after transplantation (*n* = 5–6 per group). **(I)** Donor chimerism of GFP^+^ donor in each transplanted group (*n* = 5–6 per group). **(J)** Percentage of non-erythroid cells (CD71^−^Ter-119^−^) within GFP^+^ donor cells for each transplanted group (*n* = 5–6 per group). **(K)** Percentage of GFP^+^ cells within total donor megakaryocytes (CD41^+^CD42d^+^; *n* = 5–6 per group). **(L)** Percentage of erythroid cells (CD71^+^ and/or Ter-119^+^) within GFP^+^ donor cells for each transplanted group (*n* = 5–6 per group). **(M)** Percentage of CD71^+^ cells within GFP^+^ donor cells in the peripheral blood for each transplanted group (*n* = 3 per group). Bars represent mean values. Error bars represent ± SD; *, P < 0.05; **, P < 0.01; ***, P < 0.001.

We next wondered whether we could also use the glycotyping approach to isolate a similar subset of cells we identified in the MEP population from a more heterogeneous cell population. We therefore focused on the entire c-kit^+^ cell population of HSPCs and sorted cells into HS3A8-high (top 50%) or HS3A8-low (bottom 50%) populations (Fig. 3 A). HS3A8-high c-kit^+^ HSPCs exhibited nearly fourfold lower total colony forming potential as compared to HS3A8-low c-kit^+^ HSPCs (Fig. 3 B). Furthermore, HS3A8-high c-kit^+^ cells generated mostly monocytic and erythroid colonies, while HS3A8-low c-kit^+^ cells showed increased immature GEMM (granulocyte, erythrocyte, monocyte, and megakaryocyte) colonies and significant enrichment in granulocytic colonies (Fig. 3 C). Importantly, functional megakaryocyte colony forming potential was almost completely restricted to HS3A8-low c-kit^+^ HSPCs (Fig. 3 D). To test whether the HS present on HS3A8-high c-kit^+^ cells was necessary for the differentiation down the erythroid lineage, we removed surface HS from c-kit^+^ HSPCs by digestion with a heparinase cocktail. We observed decreased colony forming potential for both erythroid BFU-E and CFU-Es, suggesting that HS is cell-autonomously required for the development of erythroid cell populations (Fig. 3, E and F).

To assess the functional capacity of HS3A8 scFv-defined cell populations in vivo, we performed competitive transplantation assays using mixtures of HS3A8 or MPB49 (control group) sorted bone marrow cells from wild-type (GFP^−^) mice and mice with GFP expression driven by a ubiquitous promotor (UBC-GFP^+^; Fig. 3 G). While the control (MPB49 mock-sorted) group and the HS3A8-sorted experimental groups resulted in similar total engraftment (Fig. 3 H), transplanted HS3A8-low GFP^+^ cells showed significantly increased overall hematopoietic reconstitution, while HS3A8-high GFP^+^ cells displayed diminished capacity for reconstitution (Fig. 3 I). Interestingly, GFP^+^ cells reconstituted from the HS3A8-low GFP^+^ transplant group showed a significant increase in non-erythroid cells compared to HS3A8-high GFP^+^ transplanted cells (Fig. 3 J). Conversely, cells from the HS3A8-high GFP^+^ transplant displayed a two- to threefold lower capacity to reconstitute megakaryocytes (Fig. 3 K) and a substantially greater capacity to reconstitute erythroid cells in the bone marrow (P = 0.051; Fig. 3 L). Additionally, the HS3A8-low GFP^+^ transplant group gave rise to ∼50% fewer peripheral blood reticulocytes (CD71^+^; Fig. 3 M). Collectively, these results demonstrate that glycotyping based on high vs. low binding of the HS3A8 scFv can distinguish HSPC subpopulations with distinct functional properties in vitro and in vivo.

### HS glycotyping separates molecularly distinct HSPCs and their lineages

**Figure S3.**
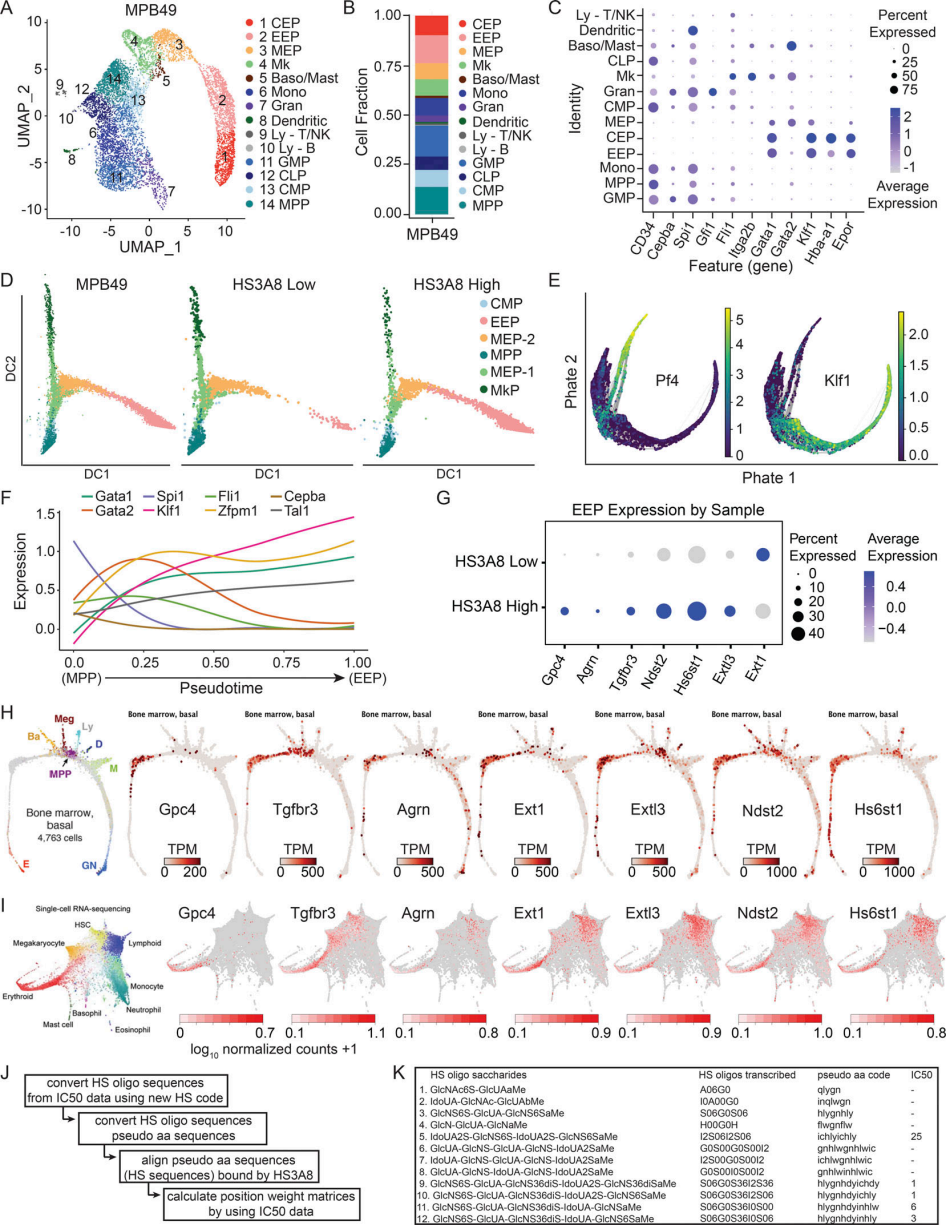
**Molecular signatures of HSPCs purified by HS scFvs, a heparan sulfate gene signature within the erythroid lineage, and determination of HS scFv binding characteristics. (A)** UMAP visualization of scRNA-seq of MPB49 sorted HSPCs segregated into transcriptionally distinct clusters. This is the control corresponding to Fig. 4 A. CEP, committed erythroid progenitor; Mk, megakaryocyte progenitor; Baso/Mast, basophil/mast cell progenitor; Mono, monocyte progenitor; Gran, granulocyte progenitor; Ly-T/NK, lymphocyte–T/natural killer cell progenitor; Ly-B, lymphocyte–B cell progenitor; CLP, common lymphoid progenitor. **(B)** Fraction of cells within each transcriptionally distinct cluster in MPB49 sorted HSPCs. **(C)** Dot plot indicating the frequency and expression of genes used for cluster validation using MPB49 sorted cells. **(D)** Diffusion mapping of the MPP to megakaryocyte progenitor or erythroid progenitor trajectories within MPB49, HS3A8-low, and HS3A8-high sorted cells. **(E)** PHATE visualization of the MPP to megakaryocyte/erythroid progenitor trajectory from integrated scRNA-seq data with respect to validating transit genes *Pf4* (megakaryocyte) and *Klf1* (erythroid). **(F)** Expression of validating transcriptional master regulator genes across pseudotime within the MPP to EEP trajectory. **(G)** Dot plot depicting expression of the identified HS heptad in EEPs. **(H)** SPRING plots colored by normalized expression, confirming the seven identified HS-related genes with enriched expression within erythroid differentiation from a scRNA-seq dataset (GSE89754). **(I)** Force-directed graphs colored by normalized expression, confirming the seven identified HS-related genes enriched in erythroid differentiation from additional scRNA-seq data (GSE107727). **(J)** Flowchart for the calculation of position weight matrices (PWM) from IC50 data (Dennissen et al., 2002). **(K)** Data from Dennissen et al. (2002) used to calculate the PWM for the HS epitope recognized by HS3A8, showing the sequences of HS oligos (column 1) transcribed into the hew HS code (column 2), and then transcribed into a pseudo amino acid sequence (column 3), along with the IC50 for HS3A8 expressed as μg/ml of HS oligo that inhibited binding of HS3A8 to heparin.

**Figure 4.**
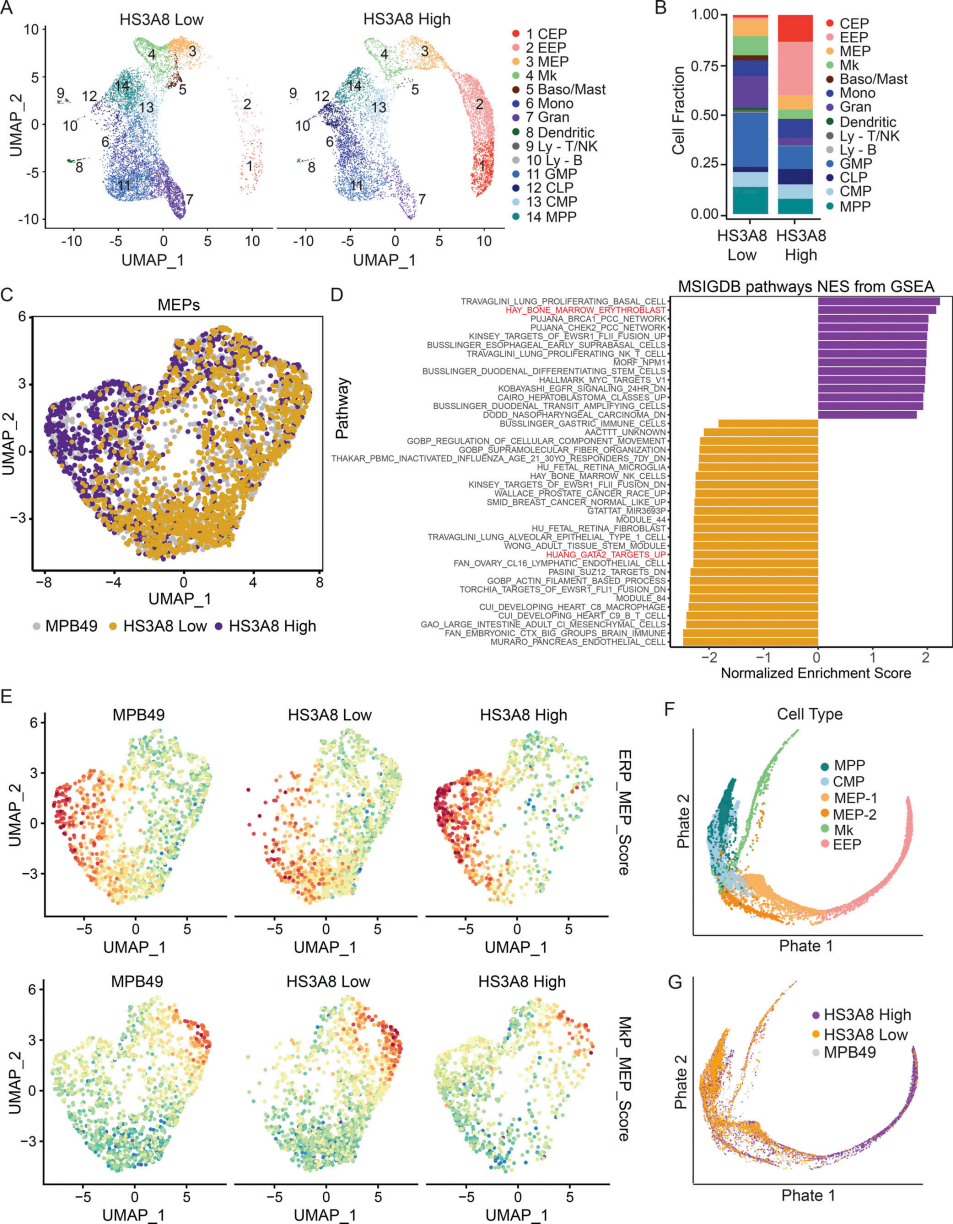
**HS3A8 scFv binding separates HSPCs into transcriptionally distinct subsets. (A)** UMAP of single-cell transcriptomes from HS3A8-low and HS3A8-high sorted Lin^−^c-kit^+^ HSPCs. Plots are colored by cluster identities (CEP, committed erythroid progenitor; Mk, megakaryocyte progenitor; Baso/Mast, basophil/mast cell progenitor; Mono, monocyte progenitor; Gran, granulocyte progenitor; Ly-T/NK, lymphocyte–T/natural killer cell progenitor; Ly-B, lymphocyte–B cell progenitor; CLP, common lymphoid progenitor). Compare also to MPB49 control UMAP in Fig. S3 A. **(B)** Quantification of the cell fraction of each transcriptionally clustered cell population within HS3A8-low and HS3A8-high sorted HSPCs. Compare also to quantification of MPB49 control-sorted cells in Fig. S3 B. **(C)** UMAP of subset MEP-like clusters (cluster 3) from MPB49, HS3A8-low, or HS3A8-high sorted cells. **(D)** GSEA analysis of HS3A8-high vs. HS3A8-low MEPs (cluster 3) isolated from scRNA-seq analysis. Purple bars indicate signatures positively enriched in HS3A8-high vs. -low MEPs and orange bars indicate signatures negatively enriched in HS3A8-high vs. -low MEPs. P < 0.05 for all signatures. **(E)** UMAP of subset MEP-like clusters colored by an assigned ERP vs. MEP score (upper panel) or MkP vs. MEP score (lower panel) generated from the top 20 up- and downregulated genes from each population (Lu et al., 2018). **(F)** PHATE projection of the MPP to EEP trajectory, colored by cluster, using integrated scRNA-seq data. **(G)** PHATE visualization of MPB49, HS3A8-low, and HS3A8-high sorted HSPCs overlaid onto the MPP to EEP trajectory.

To dissect HS-based partitioning, we sorted HS3A8-high, HS3A8-low, and MPB49 (mock-sorted, representing total) from a heterogenous pool of Lin^−^c-kit^+^ HSPCs and performed single-cell RNA-seq (scRNA-seq). Dimensionality reduction of scRNA-seq data using uniform manifold approximation and projection (UMAP) faithfully segregated single cells into a continuum of various lineages from the MPB49 sorted sample (Fig. S3, A–C). Remarkably, HS3A8-low sorted HSPCs were almost completely devoid of cells exhibiting an erythroid transcriptional profile, while these same clusters were enriched in HS3A8-high sorted HSPCs (Fig. 4, A and B). Conversely, the HS3A8-low sample was enriched for cells with megakaryocyte, granulocyte, and GMP gene signatures, as compared to the HS3A8-high sorted sample (Fig. 4, A and B). Importantly, HS3A8 binding was able to further resolve the MEP cluster (cluster 3) into two transcriptionally distinct populations, and analysis of these MEPs revealed clear functional divergence on the basis of HS3A8 binding (Fig. 4 C). Unbiased GSEA of the complete set of Molecular Signatures Database pathways revealed a GATA2 target signature (required for megakaryopoiesis; Huang et al., 2009) enriched in HS3A8-low MEPs and an erythroid-related gene signature enriched in the HS3A8 high-MEPs among the top hits (Fig. 4 D). We, therefore, hypothesized that differential erythroid vs. megakaryocytic potential drove the HS3A8-high vs. -low separation within the bipotent MEPs. To further characterize this lineage bias, enrichment signatures were derived from previously established gene expression data using the top 20 upregulated genes in highly fractionated erythroid progenitors (ERPs) and megakaryocyte progenitors (MkPs), each compared to baseline MEPs (Lu et al., 2018). Visualizations of UMAP embeddings colored by normalized module scores illustrated a clear MkP propensity within HS3A8-low sorted cells and ERP propensity within HS3A8-high MEPs (Fig. 4 E).

To obtain insight into the temporal expression of HS modification patterns along distinct cell fate trajectories, we employed trajectory inference using PHATE (Potential of Heat-diffusion for Affinity-based Transition Embedding; Moon et al., 2019) and diffusion mapping to query the expression of HS modifications as multi-potent progenitors (MPPs) transition into megakaryocyte or erythroid lineages (Fig. 4 F and Fig. S3, D–F). Interestingly, we found that HS3A8-low and HS3A8-high cells diverged at the MEP level and upon commitment to the megakaryocyte (HS3A8-low dominant) or early erythroid (HS3A8-high dominant) stages (Fig. 4 G). These data demonstrate the discriminatory power of HS glycotyping at single-cell resolution and pinpoint the differences in lineage commitment of HS3A8-high vs. HS3A8-low HSPC to the immunophenotypically defined MEP stage.

**Figure 5.**
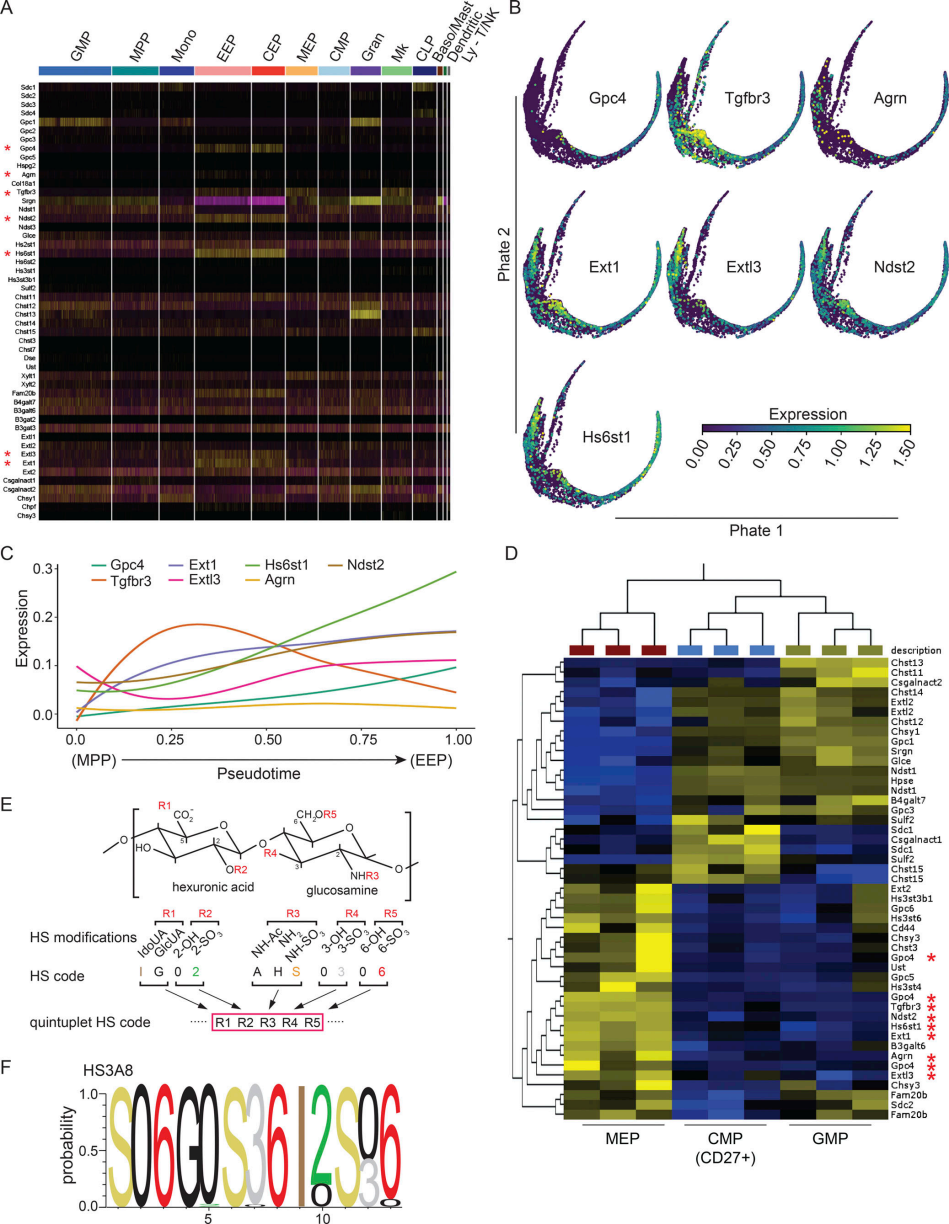
**Expression dynamics of a heptad of HS-related genes enriched in erythroid populations and binding characteristics of the HS3A8 scFv. (A)** Heatmap of the expression of HS-related genes within clustered cell populations. Asterisks indicate genes uniquely enriched in the erythroid arm of hematopoiesis. Different cell populations are color coded. CEP, committed erythroid progenitor; Mk, megakaryocyte progenitor; Baso/Mast, basophil/mast cell progenitor; Mono, monocyte progenitor; Gran, granulocyte progenitor; Ly-T/NK, lymphocyte–T/natural killer cell progenitor; CLP, common lymphoid progenitor). **(B)** PHATE visualization of *Gpc4*, *Tgfbr3*, *Agrn*, *Ext1*, *Extl3*, *Ndst2*, and *Hs6st1* expression within the MPP to EEP trajectory. **(C)** Expression of the identified HS-related gene heptad across pseudotime within the MPP to EEP trajectory. **(D)** Differentially expressed HS-related genes within CMP (CD27^+^), GMP, and MEP populations by microarray (GSE33937). Red asterisks indicate probes of the seven HS-related genes previously identified from scRNA-seq data. Yellow signifies up-regulation and blue down-regulation. **(E)** A quintuplet code that defines possible modifications on an HS disaccharide allows continuous coding of HS sequences (Townley and Bülow, 2018). The five positions that can be modified in a HS disaccharide are shown. 11 digits can describe a HS disaccharide in a quintuplet code. IdoA: iduronic acid, GluA: glucuronic acid. **(F)** Position weight matrix describing the presumptive HS epitopes bound by the HS3A8 scFv, calculated from published competitive ELISA data (Dennissen et al., 2002) using the quintuplet code. For details on calculations, see Materials and methods and Fig. S3, J and K.

To discern HS-associated genes underlying this process, we screened our composite scRNA-seq data for the expression of known HS proteoglycans and glycosaminoglycan synthesis and modifying enzymes across different subsets of hematopoietic progenitors (Fig. 5 A; Fernández-Vega et al., 2015; Crespo et al., 2018). We identified a heptad of genes (*Gpc4*, *Tgfbr3*, *Agrn*, *Ext1*, *Extl3*, *Ndst2*, and *Hs6st1*) whose expression was significantly enriched within MEPs compared to other progenitors and in erythroid populations (Fig. 5 A). Upon mapping expression of these genes onto our trajectory analysis, we found that they exhibited unique expression dynamics during the transition from progenitor to the committed erythroid stage (Fig. 5, B and C). Specifically, HS modifying enzymes, including the *Ndst2*/*N*-deacetylase-*N*-sulfotransferase and the *Hs6st1*/HS 6-*O*-sulfotransferase as well the *Gpc4*/Glypican and *Agrn*/Agrin HSPG core proteins were significantly enriched in the early erythroid progenitor (EEP) fraction (Fig. 5, B and C; and Fig. S3 G). Microarray data of hematopoietic progenitors illustrated overexpression of this HS-related heptad in MEPs as compared to CMP and GMP populations (Fig. 5 D). In further support, we found consistent enrichment of the same HS-related heptad in the erythroid populations in two publicly available scRNA-seq datasets (Fig. S3, H and I). Since we find that HS is required for erythroid differentiation, these findings suggest that this heptad of HS genes is functionally important.

Given the ability of HS3A8 to segregate hematopoietic cells into functionally and molecularly distinct populations, we wanted to gain insight into the HS epitope recognized by the HS3A8 scFv. To this end, we utilized IC50 data from competitive ELISA experiments (Dennissen et al., 2002) with 12 different HS oligosaccharides of defined sequence that measured inhibition of HS3A8 scFv binding to heparin (Fig. S3, J and K). Using this data, we generated a position weight matrix of the HS epitope recognized by the HS3A8 scFv (akin to transcription factor binding sites in DNA; Fig. 5, E and F). Intriguingly, *N*-sulfation and 6-*O*-sulfation, which can be introduced by the HS modifying enzymes *Ndst2*/*N*-deacetylase-*N*-sulfotransferase and the *Hs6st1*/HS 6-*O*-sulfotransferase, respectively, feature prominently in the putative HS epitope recognized by HS3A8 (Fig. 5 F). Therefore, the HS epitope recognized by HS3A8 on EEPs reflects the expression profile of corresponding HS modifying enzymes in this cell population.

### Human erythroid and megakaryocyte differentiation show similar and dynamic HS modification patterns

**Figure 6.**
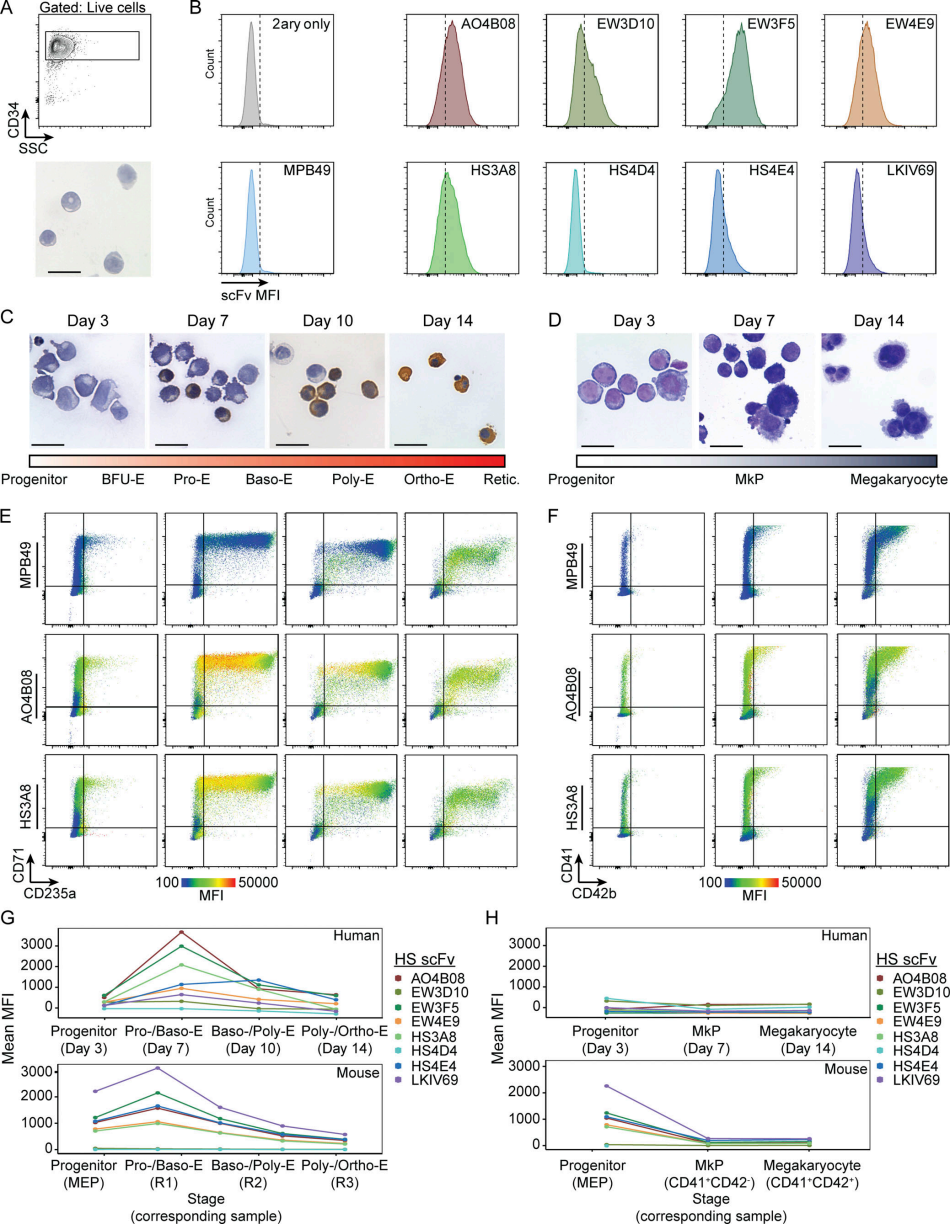
**HS scFv binding patterns within human megakaryocyte and erythroid differentiation reveal dynamically and temporally shared HS-modification patterns. (A)** Representative FACS density plot (upper panel) and morphology (May-Grunwald-Giemsa stain; lower panel) of human CD34^+^ cells. Scale bar indicates 20 μm. **(B)** Histograms of HS scFv binding to human CD34^+^ cells (donor #1). **(C)** Cell morphological time course of experimental in vitro erythroid differentiation of human CD34^+^ cells. A scheme bar indicating the distinct erythroid differentiation stages is included. Pro-E, proerythroblast; Baso-E, basophilic erythroblast; Poly-E, polychromatic erythroblast; Ortho-E, orthochromatic erythroblast. Scale bar indicates 20 μm. **(D)** Cell morphological time course of experimental in vitro megakaryocytic differentiation of human CD34^+^ cells. A scheme bar indicating distinct megakaryocytic differentiation stages is included. Scale bar indicates 20 μm. **(E)** Flow cytometry plots of HS scFv signal overlaid on the erythroid differentiation markers CD71 and CD235a during day 3, 7, 10, and 14 of erythroid differentiation (donor #1). The color coding of scFv-binding MFI is shown at the bottom. **(F)** Heatmap of HS scFv signal overlaid on the megakaryocyte differentiation markers CD41 and CD42b during day 3, 7, and 14 of megakaryocyte differentiation (donor #1). The color coding of scFv-binding MFI is shown at the bottom. **(G)** Binding of HS scFvs in stage-matched human (upper panel, donor #1) and mouse (lower panel) erythroid-lineage cells. Pro-E, proerythroblast; Baso-E, basophilic erythroblast; Poly-E, polychromatic erythroblast; Ortho-E, orthochromatic erythroblast. **(H)** Binding of HS scFvs in stage-matched human (upper panel, donor #1) and mouse (lower panel) megakaryocytic-lineage cells.

**Figure S4.**
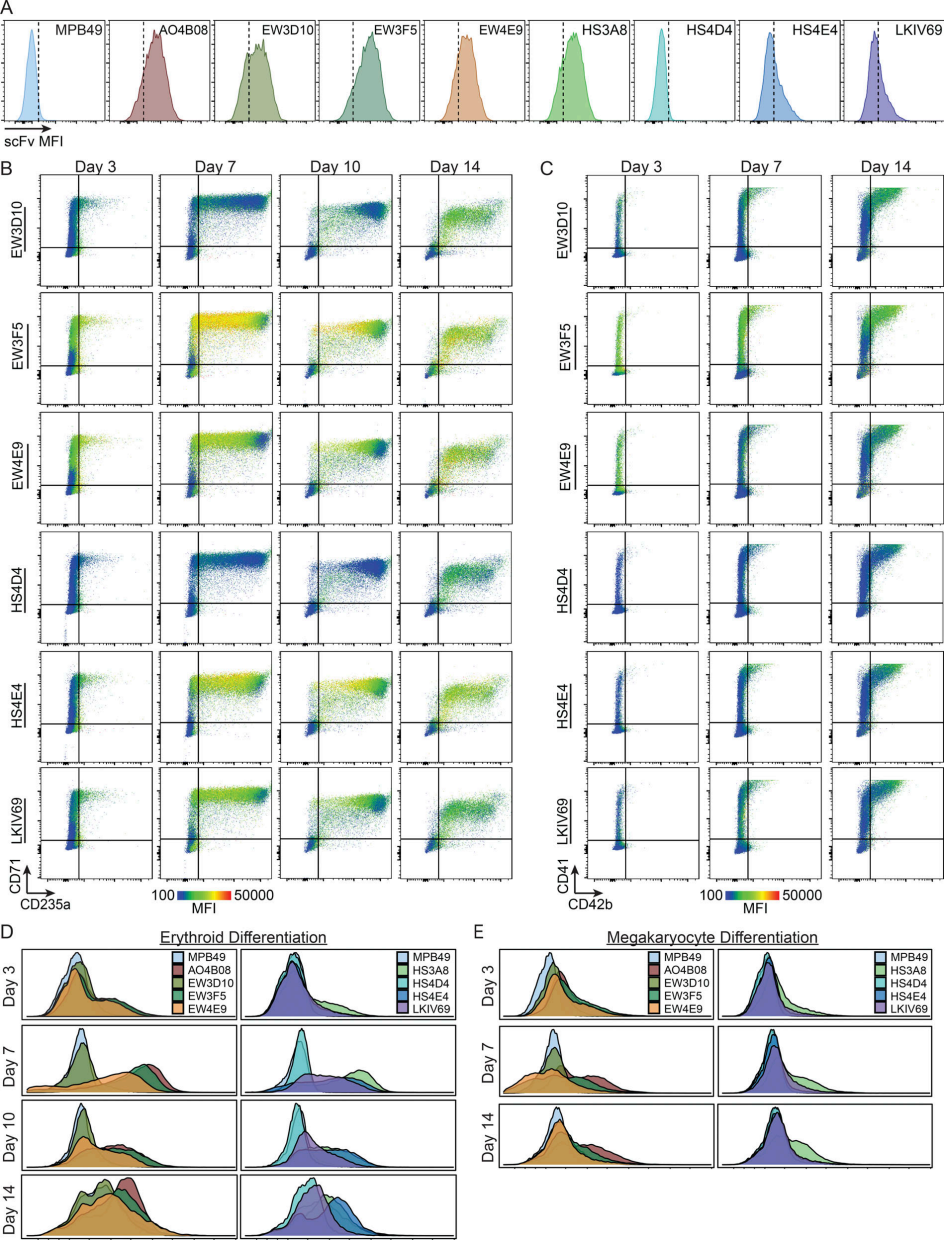
**Dynamic patterning of HS modifications within human erythroid and megakaryocyte differentiation. (A)** Histograms of HS scFv binding signal in human CD34^+^ cells from mobilized peripheral blood (donor #2). **(B)** Flow cytometry plots of HS scFv signal overlaid on the erythroid differentiation markers CD71 and CD235a during day 3, 7, 10, and 14 of erythroid differentiation (donor #1). **(C)** Flow cytometry plots of HS scFv signal overlaid on the megakaryocyte differentiation markers CD41 and CD42b during day 3, 7, and 14 of megakaryocyte differentiation (donor #1). **(D)** Distribution of the binding of each HS-specific scFv during day 3, 7, 10, and 14 of erythroid differentiation (donor #1). **(E)** Distribution of the binding of each HS-specific scFv during day 3, 7, and 14 of megakaryocyte differentiation (donor #1).

**Figure S5.**
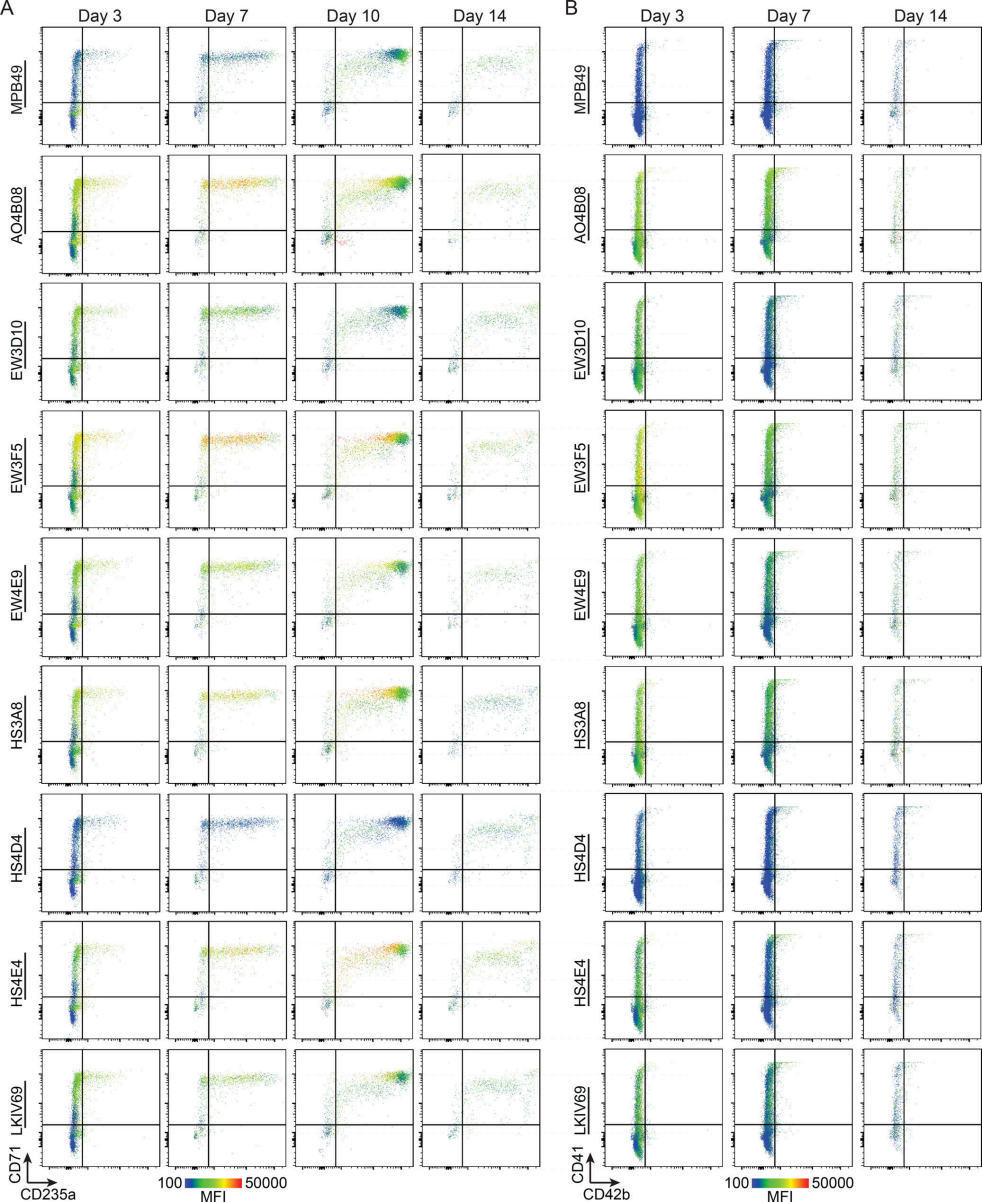
**Dynamic patterning of HS modifications within human erythroid and megakaryocyte differentiation (donor #2). (A)** Flow cytometry plots of HS scFv signal overlaid on the erythroid differentiation markers CD71 and CD235a during day 3, 7, 10, and 14 of erythroid differentiation (donor #2). **(B)** Flow cytometry plots of HS scFv signal overlaid on the megakaryocyte differentiation markers CD41 and CD42b during day 3, 7, and 14 of megakaryocyte differentiation (donor #2).

We next investigated the portability of the glycotyping approach to interrogate HS modification patterns within human progenitors and their subsequent differentiation into megakaryocyte and erythroid lineages. Interestingly, binding patterns of the HS scFv panel to human CD34^+^ HSPCs (i.e., their glycotypes) were similar to glycotypes observed in mouse HSPCs (Fig. 6, A and B; and Fig. S4 A). When we performed megakaryocytic and erythroid differentiation assays of human CD34^+^ cells in vitro (Fig. 6, C and D), we found the temporal and dynamic binding patterns of HS scFvs during human erythroid commitment (Fig. 6 E; and Fig. S4, B and D; and Fig. S5 A) as well as megakaryocyte differentiation (Fig. 6 F; and Fig. S4, C and E; and Fig. S5 B) to resemble those observed in the murine system. Mapping scFv binding of similarly staged cell populations between human (data from Fig. 6, E and F, and Fig. S4, B and C) and mouse (data from Fig. 1, D and F) showed a shared pattern of HS scFv binding dynamics, in which binding of several scFvs increased upon early erythroid commitment and decreased during terminal differentiation, while megakaryocytic differentiation was characterized by relatively diminished and stable levels of HS scFv binding (Fig. 6, G and H). These data suggest a shared dynamic patterning of HS modifications recognized by HS scFvs during erythroid commitment in mouse and humans and consistently low levels of surface HS on megakaryocytic cells (at least as far as recognized by the eight scFvs used here).

**Figure 7.**
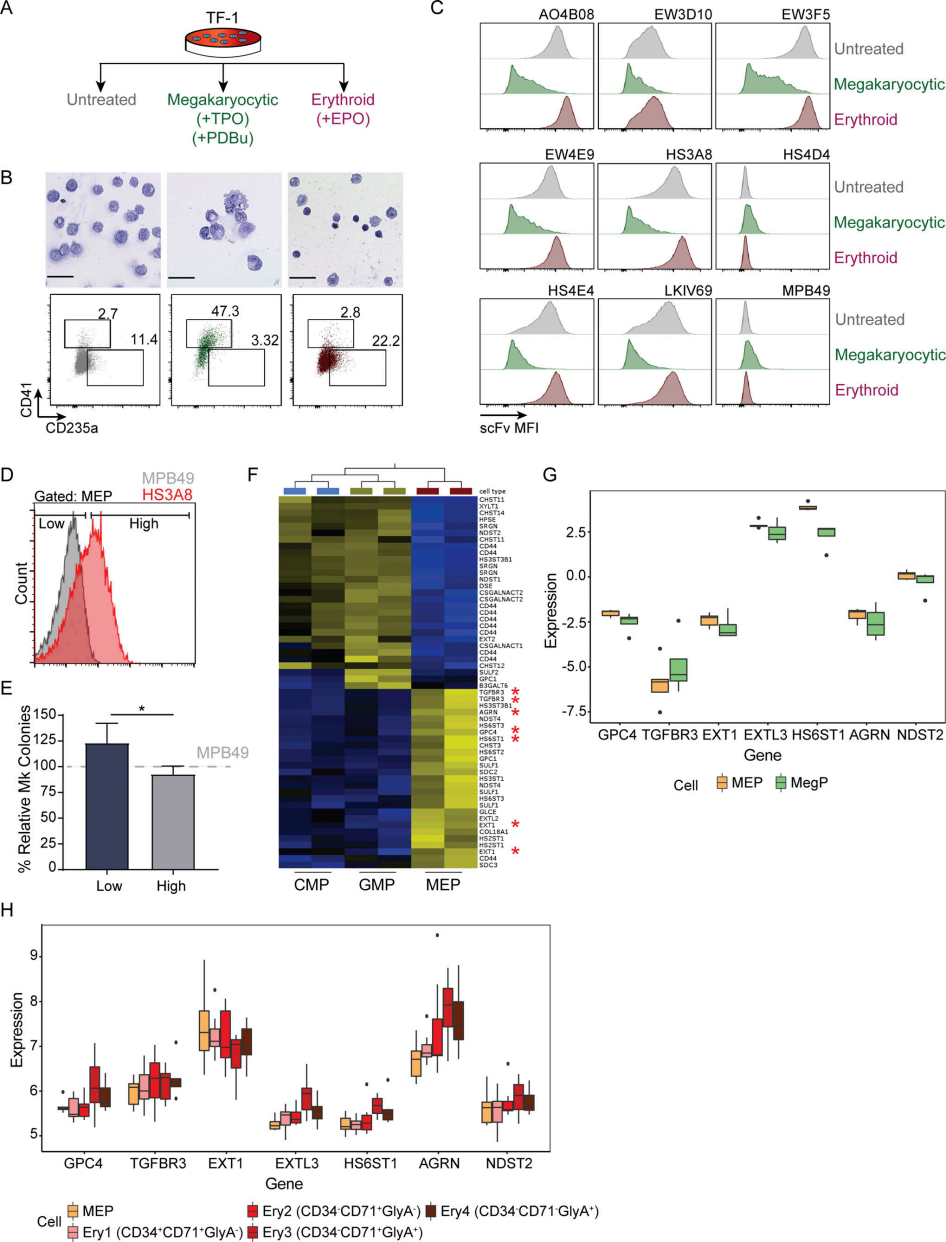
**HS modification alteration of the TF-1 cell line upon erythroid and megakaryocyte differentiation, and assessment of functional megakaryocytic output of human HS3A8-sorted MEPs. (A)** Schematic of TF-1 differentiation into erythroid and megakaryocytic cells. TPO, thrombopoietin; PDBu, phorbol 12,13-dibutyrate; EPO, erythropoietin. **(B)** Morphological (upper panels) and immunophenotypic (lower panels) validation of erythroid and megakaryocyte differentiation of TF-1 cells. Scale bar indicates 50 μm. **(C)** Representative histograms of HS scFv binding of TF-1 cells in parental (gray), megakaryocyte (green), and erythroid (red) differentiation conditions (*n* = 3). **(D)** Sorting of human MEPs (Lin^−^CD34^+^CD38^+^CD123^−^CD45RA^−^) into low (bottom 35–45%) and high (top 35–45%) populations based on binding of HS3A8. The gray histogram shows the MPB49 (non HS-binding scFv) control. **(E)** Relative percentage of megakaryocyte (Mk) colonies in HS3A8-high/-low sorted human MEPs as compared to MPB49 mock-sorted controls (*n* = 6). **(F)** Significantly differentially expressed HS-related genes within human CMP, GMP, and MEP populations from GSE19599. Red asterisks indicate probes of the HS-related genes of the previously identified HS heptad from scRNA-seq data in mice. Yellow signifies up-regulation and blue down-regulation. **(G)** Expression of HS-related gene heptad in human MEP and megakaryocyte-committed progenitors (MegP) by microarray (GSE77439). **(H)** Expression of HS-related gene heptad in human MEP and various stages of erythroid differentiation (Ery1–4) by microarray (GSE24759). Bars represent mean values of biological replicates. Error bars represent ± SD; *, P < 0.05.

Finally, we explored HS scFv binding along erythroid and megakaryocyte differentiation. We first modeled the dynamics of HS scFv binding during lineage commitment by implementing a longitudinal differentiation assay utilizing bipotent (erythroid/megakaryocytic) human TF-1 cells. Interestingly, loss of scFv binding in cells during megakaryocytic differentiation and increased binding of several HS scFvs in cells during erythroid differentiation was evident (Fig. 7, A–C). Functionally, human MEPs sorted based on their HS3A8 binding displayed consistent differences in megakaryocyte colony-forming potential between HS3A8-high vs. -low binding populations (Fig. 7, D and E). Additionally, analysis of publicly available gene expression data from FACS-sorted human progenitors revealed that five of the seven HS-related genes from our heptad identified in mice were also enriched in human MEPs compared to CMP and GMP populations (Fig. 7 F). Indeed, when investigating the expression of our identified HS-related gene heptad in MEPs as compared to committed megakaryocyte progenitors or increasingly committed erythroid populations, we observed decreased expression in six of seven genes during megakaryocyte commitment, and a consistent, dynamic patterning of six of seven genes during erythroid differentiation (Fig. 7, G and H).

Taken together, these data suggest that HS modification patterns and dynamics in hematopoietic progenitors are similar between mice and humans, particularly during megakaryocyte and erythroid differentiation. Moreover, these data establish HS glycotyping as an immunophenotypically, functionally, and molecularly viable approach for the further characterization, separation, and isolation of distinct hematopoietic and possibly other cell populations.

## Discussion

**Figure 8.**
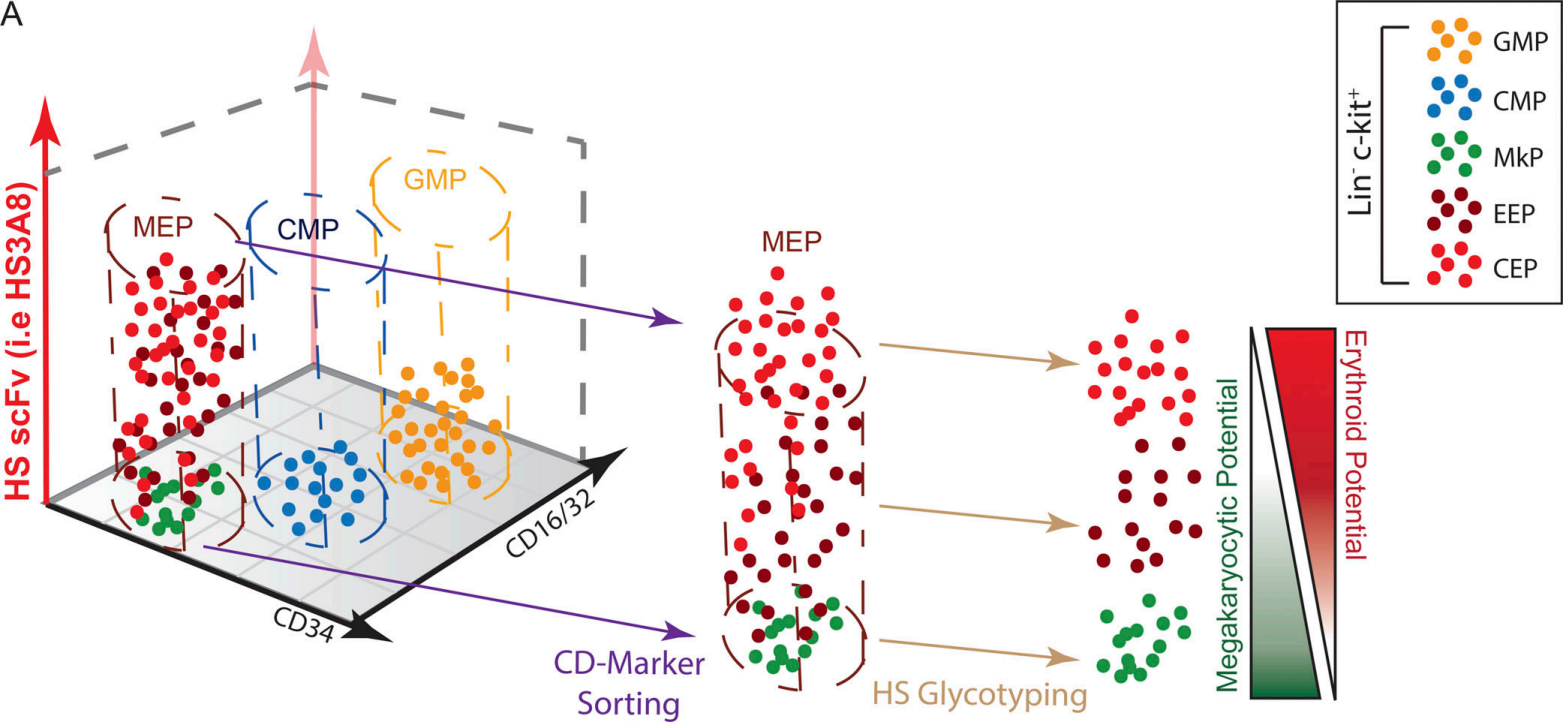
**Overview of the orthogonal application of HS glycotyping for the separation of distinct cell populations. (A)** Schematic depicting the characterization and separation of immunophenotypic murine hematopoietic progenitor populations (Lin^−^c-kit^+^Sca-1^−^) by conventional CD markers (i.e., CD34 and CD16/32) and by HS glycoptyping with scFvs (e.g., HS3A8). HS glycotyping provides an additional dimension of separating heterogenous cell populations (i.e., MEPs) into further refined and distinct functional and molecular subsets. CEP, committed erythroid progenitor.

The detection of cell surface proteins by flow cytometry has revolutionized the ability to characterize and isolate cells from heterogeneous tissues such as blood and bone marrow (Akashi et al., 2000; Zhang et al., 2003; Oguro et al., 2013; Gabius et al., 2015). Yet, limitations in flow cytometry–based methods remain, including expression of only restricted subsets of CD-classified surface proteins and frequent inability to directly isolate specific cell types (Mayle et al., 2013). Moreover, some proteins recognized by CD markers (e.g., CD44) reveal differences in their respective decoration with glycosaminoglycans (Bennett et al., 1995; Jackson et al., 1995), underscoring the need to obtain higher resolution using the glycotyping approach. Importantly, single-cell sequencing approaches reveal a much higher cellular diversity than what can currently be distinguished by CD markers alone (Olsson et al., 2016; Lindahl et al., 2017; Karamitros et al., 2018; Chen et al., 2019; Wheat et al., 2020; Schwenger and Steidl, 2021), but these techniques are invariably destructive, precluding the isolation of viable cell populations. Therefore, the extraordinarily rich ensemble of HS modification patterns rather than the limited set of core proteins that bear them (Turnbull et al., 2001; Esko and Selleck, 2002; Lindahl and Li, 2009) provides an exciting new avenue for the phenotypic and functional classification of cells. Importantly, our approach facilitates the isolation of unique and rare cellular subsets that cannot be distinguished and isolated using existing approaches (Fig. 8). Given the discrimination observed with a limited panel of only eight HS-directed scFvs, we anticipate even greater opportunities to define distinct functional cell populations, including more mature ones, through expansion and combinatorial use of HS-specific scFvs in the future.

The glycotyping approach described in this study revealed expression patterns and functional associations of HS modification patterns (i.e., unique glycotypes) in distinct subsets of hematopoietic progenitors, specifically within megakaryocyte–erythroid lineages. While we primarily focused our studies on immunophenotypically defined MEPs and downstream erythroid progenitors, elegant prior work characterized and proposed pre-megakaryocyte–erythrocyte progenitors (PreMegE) progenitors based on several additional CD markers (Pronk et al., 2007). We predict evaluation of HS patterns in the aforementioned context and other proposed CD marker-based schemes to further subdivide various HSPC fractions in the future.

In our study, the temporal and dynamic expression of HS modification patterns within differentiating erythroid cells were remarkably similar in both primary mouse and human HSPCs, indicating a substantial degree of conservation. scRNA-seq experiments revealed that the glycotype of the terminally differentiating erythroid lineage is distinctly marked by increased expression of a heptad of HS-related genes (*Gpc4*, *Agrn*, *Tgfbr3*, *Ext1*, *Extl3*, *Ndst2*, and *Hs6st1*), implicating these genes as well as *N*- and 6-*O*-sulfated HS as important contributors to erythroid identity and development. In support of this conclusion, *Hs6st1*-deficient mice lacking the enzyme for Heparan Sulfate 6-*O*-Sulfotransferase 1 (Hs6st1) display embryonic lethality accompanied by a complete absence of placental erythroid cells (Habuchi et al., 2007). Moreover, in vitro differentiation of embryonic stem cells lacking HS in hematopoietic cells could be rescued by exogenous addition of *N*- and 6-*O*-sulfated HS (Holley et al., 2011). Our findings reveal a specific and shared heparan sulfate program that is established during megakaryocytic-erythroid specification and fully activated in terminal erythroid differentiation.

In conclusion, this study provides proof-of-concept for HS glycotyping within mouse and human hematopoietic tissues, affording a new platform for the prospective isolation, characterization, and identification of cellular subsets. While we have focused on hematopoiesis in this study, we expect this approach to have far-reaching applicability in a diversity of species and tissues, spanning a broad range of physiological conditions and disease states. Overall, HS glycotyping offers a novel orthogonal method for the identification and purification of cells with substantial biological, diagnostic, and therapeutic potential.

## Materials and methods

### Mice

C57Bl/6 wild-type (CD45.2), B6.SJL-*Ptprc*^*a*^ *Pepc*^*b*^/BoyJ (CD45.1), and C57BL/6-Tg(UBC-GFP)30Scha/J (CD45.2) mice were purchased from Jackson Labs. All mice were age- (6–12 wk) and sex-matched for all experimental studies. Mice were housed in a special pathogen–free (SPF) barrier facility. All animal experiments were performed in compliance with institutional guidelines and approved by the Animal Institute Committee of the Albert Einstein College of Medicine (#00001099).

### HS scFv production

HS scFvs were expressed from a modified pNYCOMPS C-term vector and transformed into BL21(DE3)-pRIL competent bacteria. Overnight cultures of sequence-validated clones were grown in 2XYT media for 90 min at 37°C with shaking to an OD600 of 0.4–0.6 before induction. Induction was performed at 30°C for 3 h with 0.1–0.25 mM IPTG, and cells were then pelleted and frozen at −80°C until lysis. Pellets were resuspended in increments of Buffer A (50 mM Hepes, pH 7.4, 20% sucrose, 1 mM EDTA, and DNase [10 μg/ml]) up to 5% of culture volume. The suspension was incubated at 4°C or on ice for 30–45 min and then spun at 30,000 *g* for 60 min. The supernatant was transferred and spun again for 30 min before being diluted 5X in Buffer B (20 mM Hepes, pH 7.4, 500 mM NaCl, 20 mM imidazole, and 10% glycerol). The scFv-containing solution was purified using an AKTAxpress (GE Life Sciences), where diluted solution was loaded on a 5-ml Ni affinity column and washed with 1 column volume of Buffer B. Gradient elution was then performed using 95–99% Buffer C (20 mM Hepes, pH 7.4, 500 mM NaCl, 200 mM imidazole, and 10% glycerol) and 10-ml aliquots of eluted protein were collected. Contaminants were removed by fractionation on a S-75 gel filtration column (GE Life Sciences) using Buffer D (20 mM Hepes, pH 7.4, 150 mM NaCl, 10% glycerol). Antibodies were brought to a concentration of 1 mg/ml and stored in aliquots of 50 or 100 μl at −80°C until time of use. Once thawed, aliquots of antibodies were stored at 4°C for a maximum of 3 mo.

### SDS-PAGE and coomassie staining

1 μg of each purified HS scFv was added to 1× sample buffer, heated to 95°C for 5 min, and then run on a NuPAGE 4–12% gradient gel (Invitrogen). The gel was then stained with SimplyBlue SafeStain Coomassie G-250 stain (Invitrogen) following the manufacturer’s protocol.

### Cell lines and human samples

The 32D, TF-1, HEL 92.1.7, and K-562 cell lines were purchased from the American Type Culture Collection and grown in IMDM or RPMI medium supplemented with 10% FBS and 1% penicillin/streptomycin. The 32D cell line was supplemented with 10% WEHI-3B–conditioned media, while the TF-1 cell line was grown in 4 ng/ml GM-CSF. The MOLM-13 and MOLM-14 cell lines were obtained from the German Collection of Microorganisms and Cell Cultures and grown in RPMI supplemented with 10% FBS and 1% penicillin/streptomycin. The murine erythroleukemia (MEL) and ES-EP cell lines were a kind gift from Dr. Arthur Skoultchi (Albert Einstein College of Medicine). MEL cells were grown in DMEM medium supplemented with 10% FBS and 1% penicillin/streptomycin, while ES-EP cells were grown in STEM MAX Media with 10% FBS and 1% penicillin/streptomycin, supplemented with glutamine (2 mM), β-mercaptoethanol (55 μM), SCF (100 ng/ml), dexamethasone (10^−6^M), erythropoietin (2 U/ml), and insulin-like growth factor-1 (40 ng/ml). Human mobilized CD34^+^ cells were obtained from commercial sources (Stemcell Technologies) or Montefiore Medical Center (IRB# 2008-842).

### Heparinase digestion

Heparinase I (H2519), Heparinase II (H6512), and Heparinase III (H8891) were purchased from Sigma-Aldrich, and stocks were prepared as per manufacturer protocol at a concentration of 10 U/ml. Cells (3 × 10^6) were digested with a cocktail of Heparinase I, II, and III (0.5 U each) in IMDM for 4 h at 37°C with agitation every 30 min. Cells were washed twice with PBS and then stained and analyzed by flow cytometry. For colony assay experiments, cells were digested with a cocktail of Heparinase I, II, and III and then washed with PBS before being plated into methylcellulose containing vehicle (undigested) or the Heparinase I, II, and III cocktail (0.5 U each).

### Flow cytometry

All antibodies used were purchased from eBioscience or BioLegend unless otherwise stated. Anti-His PE secondary antibody was from Miltenyi Biotec. Lineage selection cocktail included CD3, CD4, CD8, B220, CD19, Gr-1, Ter-119, CD11b, and Gr-1. Total bone marrow cells were isolated from tibiae, femurs, and pelvic bones by gentle crushing in PBS followed by erythrocyte lysis with ACK buffer, or isolation through Ficoll-Paque. Primary stains were performed for 30 min on ice, then washed once with 2% PBS/FBS, and a secondary stain (if needed) was performed for 25 min on ice. Cells were washed two to three times and then analyzed using either a five-laser FACS Aria II Special Order System, FACS Canto II System, or LSR II System containing a yellow laser (BD LSR II-Y; Becton Dickinson). All cell sorting was performed on either a five-laser FACS Aria II Special Order System or a Beckman Coulter Moflo Astrios EQ System. A secondary only control of Anti-His PE (with no primary scFv stain) was used, along with the MPB49 which acts as a negative control scFv. Analysis of FACS data was performed using BD FACSDiva (Becton Dickinson) and FlowJo (Tree Star) software.

### Methylcellulose colony forming assays

FACS-purified cells were sorted into FBS, spun down at 300 *g*, and then plated into either methylcellulose, BFU-E, or MegaCult media as per the manufacturer’s protocol. For methylcellulose colony assays, FACS-purified c-kit^+^ (5,000 per well) bone marrow cells were plated in HSC007 methylcellulose (R&D Systems). For BFU-E assays, FACS-purified MEP (3,500 per well) or c-kit^+^ cells treated with vehicle or Heparinase I, II, III (3,500 per well) were sorted and plated in HSC006 methylcellulose (R&D Systems) supplemented with 50 ng/ml SCF, 20 ng/ml IL-3, 20 ng/ml IL-6, and 10 U rhEPO. Colonies were then counted 7 d later. For MegaCult assays, FACS-purified c-kit^+^ (3,500 per well) or MEP (3,500) mouse bone marrow cells were sorted and plated in MegaCult media as per manufacturer protocol (STEMCELL Technologies). Human CD34^+^ cells from mobilized peripheral blood were allowed to recover in StemSpan II media with cytokines (SCF, IL-3, Flt,IL-6, and TPO all at 50 μg/ml) for 12 h before sorting, and human MEPs (3,000–4,500 per condition) were sorted and plated in MegaCult. For mouse samples, colonies with three or more megakaryocytes were scored 8 d later based on acetylcholinesterase positivity following manufacturer’s protocol. For human samples, slides were stained with the MegaCult Human Staining Kit as per manufacturer protocol.

### Competitive transplantation

An equal number of FACS-purified c-kit^+^ cells from wild-type c57BL/6 mice and UBC-GFP mouse bone marrow were mixed and resuspended in HBSS and injected into the right femurs of lethally (1,200 Gy split dose) Pepc/BoyJ CD45.1 recipient mice at 50,000 cells per mouse. MPB49 (control) or HS3A8-high and -low sorted HSPCs from both GFP^−^ and GFP^+^ donors were oppositely mixed and transplanted (Group A: total MPB49 GFP^−^ mixed with total MPB49 GFP^+^; Group B: HS3A8-low GFP^+^ mixed with HS3A8-high GFP^−^; Group C: HS3A8-low GFP^−^ mixed with HS3A8-high GFP^+^). Recipient mice were sacrificed 2 wk after transplantation, and the bone marrow and peripheral blood of each individual mouse were isolated and analyzed by flow cytometry for chimerism. GFP levels were used as a readout of blood cell chimerism arising from transplanted progenitors.

### Cell line differentiation assays

ES-EP cells were differentiated in STEM MAX media (with FBS and penicillin/streptomycin) plus 10 U/ml of erythropoietin, 10 μg/ml insulin, and mifepristone (3 nM). After 72 h in culture, cells were washed in PBS and used for flow cytometry analysis or benzidine-hematoxylin staining. TF-1 cells were starved of GM-CSF overnight and then either maintained in full parental media or supplemented with 10 U/ml of erythropoietin (Erythroid differentiation media) or mTPO 20 ng/ml and PDBu 20 nM (megakaryocyte differentiation media). Cells were maintained in respective media for 4 d, after which cells were harvested for flow cytometry and cytospin analysis.

### Cell morphology and staining

Cytospins of cells were stained by either benzidine-hematoxylin staining as described (Wickrema et al., 1992) or using a modified May-Grunwald-Giemsa stain (Shandon Kwik-Diff Stains; Thermo Fisher Scientific).

### Real-time PCR analysis

Ficoll-processed bone marrow from mouse femurs, tibia, hips, and spine were lineage depleted, and 20,000 MEPs for each condition were sorted directly into RLT buffer. RNA was extracted using the RNAeasy micro kit (Qiagen) as per the manufacturer’s protocol, and cDNA was generated using the iScript system (BioRad). Amplification of target genes was measured using the Power SYBR Green mix (Applied Biosystems), and cDNA was amplified in a final volume of 15 μl in 96-well or 384-well microtiter plates according to the manufacturer’s recommendation. All real-time PCR experiments were performed using a ViiA7 instrument (Life Technologies) with one cycle of 50°C (2 min) and 95°C (10 min) followed by 40 cycles of amplification, with each cycle comprising the steps: 95°C (15 s) and 60°C (for 1 min). Specific amplification of the target genes was validated by melting curve analysis and Sanger sequencing, and gene expression quantification was calculated using the Pfaffl model and normalized to *Gapdh* expression levels. Primer sequences are listed in Table S3.

### Differentiation of human CD34^+^ cells

CD34^+^ cells isolated from peripheral blood of two mobilized human donors were obtained from Dr. Amit Verma upon approval of the Institutional Review Board of Albert Einstein College of Medicine (protocol 2008-842). Differentiation into megakaryocyte or erythroid cells was performed as previously described (Pineault et al., 2013; Sundaravel et al., 2015). Briefly, 2 × 10^6^ CD34^+^ cells were placed in megakaryocyte or erythroid differentiation media. On day 0, 3, 7, 10, and 14, cells were harvested for flow cytometry (1 × 10^5^ per scFv), cytospin (50,000 cells), or RNA analysis (1 × 10^5^).

### RNA-seq experiments

The RNA from FACS-purified MEPs (25,000 cells per condition) were submitted to Novogene for low-input RNA-seq analysis and sequenced on the Illumina Hi-Seq platform. Quality control was performed on the basis of error distribution along the length or reads, GC distribution, N content, base quality, and adaptor content. Reads were mapped to the mm10 transcriptome using STAR aligner (Dobin et al., 2013). Raw counts were subsequently normalized and analyzed for differential expression in R using the Bioconductor package DESeq2 (Love et al., 2014). An enrichment score was generated using the negative logarithm of the adjusted P value multiplied by the sign of the fold-change for each gene and input into pre-ranked GSEA (v4.0.2, Broad Institute; Subramanian et al., 2005). Pre-ranked gene lists were queried against standard c1-7 and hallmark Molecular Signatures Database gene lists including select erythroid and megakaryocyte gene lists.

### scRNA-seq experiments

FACS-purified HSPCs (c-kit^+^) sorted on HS scFv binding were used to prepare scRNA-seq libraries using the 10x Genomics Chromium 3′v3 kit according to manufacturer protocol (10x Genomics). Single cells were isolated using the Gemcode technology platform using barcoded droplets and paired-end reads of 150 bp. Libraries were submitted for sequencing to Novogene using an Illumina Hi-Seq platform with a target of 10,000 cells per condition. Raw sequencing data was demultiplexed, aligned, and quantified via Alevin (Patro et al., 2017). Data was further scaled, normalized, integrated, and analyzed using Seurat (Butler et al., 2018; Stuart et al., 2019). Clustering was achieved via the original Louvain algorithm, and dimensionality reduction, data visualization, and marker identification were performed using Seurat, while pseudotime analysis and diffusion maps were generated via PHATE and Scanpy (Wolf et al., 2018; Moon et al., 2019). Preranked GSEA scores were generated from the negative logarithm of adjusted P values derived from the Wilcoxon rank sum test multiplied by the sign of the fold-change. ERP vs. MEP and MkP vs. MEP scores were calculated using the top 20 up- and downregulated genes from previously published expression data, normalized to housekeeping gene expression, and mapped onto existing UMAP embeddings (Lu et al., 2018).

### GEO

Gene expression data generated from RNA-seq of MEPs sorted on HS3A8 binding are provided under accession no. GSE206672. scRNA-seq data generated from MPB49 and HS3A8-sorted c-kit^+^ HSPCs are provided under accession no. GSE206671.

### Analysis of gene expression databases

Analysis of previously published scRNA-seq data (GSE89754 and GSE107727) was done using the respective data analysis platform from each study (Dahlin et al., 2018; Tusi et al., 2018). Analysis of previously published gene expression microarray data obtained from the GEO database (GSE33937, GSE19599, GSE77439, GSE24759) was performed using the QLucore Omics Explorer software (QLucore).

### Calculation of position weight matrices

Position weight matrices were calculated based on published IC50 data from competitive ELISA experiments, which used 12 different HS oligosaccharides of defined sequence to measure the inhibition of binding of HS scFvs to heparin, and a newly devised HS coding system in which a quintuplet code of 11 digits can describe any HS oligo (Dennissen et al., 2002; Townley and Bülow, 2018). The 12 HS oligosaccharides were first transcribed using the HS coding system, and then into “pseudo amino acid” sequences (Fig. S3, J and K). Using this arbitrary step allowed alignment using ClustalW of all HS oligo sequences (in the form of amino acid sequences) that bound to the HS3A8 scFv. A multiple sequence file (MSF) with HS oligo sequences that inhibited binding of HS3A8 in proportion to the IC50 was created. To take into account different IC50 values, the IC50 previously determined for each different HS oligosaccharide sequence (Dennissen et al., 2002) was related to 100. For example, 100 was divided by the IC50 value, and that number determined the number of times a sequence was contained in the MSF. If the IC50 value was 25, the corresponding sequence was included four times in the MSF; if the IC50 was 1, the corresponding sequence was included 100 times in the MSF. Next, the MSF was input into a web-based software (https://weblogo.berkeley.edu/logo.cgi) to calculate a position weight matrix. The resulting matrix of amino acid sequences was then converted back into the HS code to yield a position weight matrix for the epitope recognized by the HS3A8 scFv. It should be noted that the 12 oligos were sufficient to create distinct position weight matrices for different HS scFv antibodies (data not shown).

### Statistics and reproducibility

Throughout this study, P values were determined by two-tailed Student’s *t* test, and error bars represent the mean ± SD of biological replicates unless otherwise indicated. Statistical analysis of group comparisons was performed using Student’s *t* test in Excel or GraphPad Prism. A value of P < 0.05 was used to determine whether a significant difference existed between two groups. All experiments were performed with a minimum of three biological replicates unless otherwise indicated. Average mean fluorescence intensity (MFI) ± SD values for all relevant experiments can be found in Tables S1 and S2.

### Online supplemental material

Fig. S1 presents data on the engineering and optimization of HS scFv’s for use in flow cytometry in cell lines and mouse bone marrow tissue. Fig. S2 shows the glycotyping of mouse bone marrow tissue and focuses on the HS scFv binding during megakaryocyte and erythroid differentiation. Fig. S3 provides additional data on the scRNA-seq and gene expression analysis of total bone marrow progenitors separated by HS3A8 binding, further identification of a heptad of HS-related genes involved in erythroid identification, and information on the proposed binding characteristics of the HS3A8 scFv. Fig. S4 shows the glycotyping of human CD34 cells from the peripheral blood of a mobilized donor at baseline and under erythroid and megakaryocyte differentiation in vitro. Fig. S5 depicts the glycotyping of human CD34 cells from the peripheral blood of a mobilized donor (donor #2) under erythroid and megakaryocyte differentiation in vitro. Table S1 lists the MFIs for all scFvs in all cell line experiments. Table S2 lists the MFIs for all scFvs in all primary mouse cell populations. Table S3 lists sequences for primers used in qRT-PCR experiments.

## Supplementary Material

Table S1 shows HS scFv binding intensity in hematopoietic cell lines.

Table S2 shows HS scFv binding intensity in murine hematopoietic populations.

Table S3 lists primer sequences for mouse gene expression analyses.
