# Supplementary material for: A glycan-based approach to cell characterization and isolation: Hematopoiesis as a paradigm
Source: J Exp Med. 2022 Sep 6;219(11):e20212552. doi: 10.1084/jem.20212552 (PMC9455685; doi:10.1084/jem.20212552)
Supplement: Table S1 — shows HS scFv binding intensity in hematopoietic cell lines. [file JEM_20212552_TableS1.docx]

| Cell Line Glycotyping |  | 32D | HEL | | K-562 | MOLM-14 | | MEL |
| --- | --- | --- | --- | --- | --- | --- | --- | --- |
|  | **scFv** | Average MFI ± SD | Average MFI ± SD | | Average MFI ± SD | Average MFI ± SD | | Average MFI ± SD |
|  | AO4B08 | 226.33 ± 45.88 | 919.00 ± 169.14 | | 1052.00 ± 113.62 | 5297.33 ± 1751.5 | | 30603 ± 15990 |
|  | EW3D10 | 220.67 ± 49.65 | 774.00 ± 172.55 | | 5292.67 ± 786.98 | 1981.67 ± 595.80 | | 1355.00 ± 204.26 |
|  | EW3F5 | 223.33 ± 48.27 | 1467.00 ± 393.91 | | 5913.33 ± 455.82 | 7746.67 ± 1634.8 | | 28730 ± 12345 |
|  | EW4E9 | 222.67 ± 50.82 | 882.67 ± 224.13 | | 2548.67 ± 707.02 | 2584.33 ± 727.96 | | 9889.00 ± 3319.2 |
|  | HS3A8 | 223.33 ± 51.05 | 802.00 ± 217.14 | | 6893.00 ± 1690.3 | 3851.67 ± 1036.3 | | 25172 ± 12803 |
|  | HS4D4 | 220.00 ± 50.48 | 171.00 ± 6.56 | | 169.67 ± 5.69 | 118.67 ± 3.512 | | 232.33 ± 29.84 |
|  | HS4E4 | 222.67 ± 50.72 | 433.00 ± 154.42 | | 858.67 ± 232.69 | 1872.33 ± 381.86 | | 16626 ± 7913.9 |
|  | LKIV69 | 224.00 ± 46.77 | 519.67 ± 132.76 | | 603.33 ± 168.59 | 1297.67 ± 228.26 | | 17233 ± 3386.2 |
|  | MPB49 | 221.67 ± 49.10 | 199.67 ± 41.59 | | 434.33 ± 88.95 | 235.00 ± 33.16 | | 294.67 ± 127.12 |
| ES-EP Differentiation |  | Parental | R1 | | R2 | R3 | | R4 |
|  | **scFv** | Average MFI ± SD | Average MFI ± SD | | Average MFI ± SD | Average MFI ± SD | | Average MFI ± SD |
|  | AO4B08 | 645.33 ± 323.36 | 614.00 ± 116.77 | | 503.00 ± 93.95 | 323.67 ± 8.33 | | 287.00 ± 44.14 |
|  | EW3D10 | 584.33 ± 125.29 | 373.00 ± 66.73 | | 319.00 ± 91.69 | 190.67 ± 70.73 | | 186.37 ± 88.60 |
|  | EW3F5 | 1692.00 ± 966.18 | 1108.33 ± 213.01 | | 848.33 ± 159.29 | 592.33 ± 17.21 | | 542.33 ± 61.99 |
|  | EW4E9 | 1036.67 ± 94.79 | 580.00 ± 18.68 | | 453.67 ± 29.74 | 277.00 ± 45.08 | | 273.33 ± 88.10 |
|  | HS3A8 | 666.33 ± 144.66 | 635.00 ± 29.51 | | 510.00 ± 30.81 | 336.33 ± 73.33 | | 309.67 ± 92.57 |
|  | HS4D4 | 112.17 ± 21.10 | 212.33 ± 58.05 | | 220.67 ± 65.25 | 95.00 ± 21.53 | | 84.77 ± 29.12 |
|  | HS4E4 | 741.67 ± 400.44 | 531.33 ± 34.08 | | 425.67 ± 44.84 | 256.67 ± 27.54 | | 245.67 ± 63.22 |
|  | LKIV69 | 2011.00 ± 562.67 | 573.00 ± 90.84 | | 418.67 ± 76.35 | 250.33 ± 16.56 | | 281.00 ± 86.37 |
|  | MPB49 | 198.67 ± 13.01 | 214.67 ± 36.47 | | 215.00 ± 68.77 | 99.07 ± 25.60 | | 88.20 ± 35.76 |
| TF-1  Differentiation |  | Parental (Untreated) | | Megakaryocyte Differentiation | | | Erythroid Differentiation | |
|  | **scFv** | Average MFI ± SD | | Average MFI ± SD | | | Average MFI ± SD | |
|  | AO4B08 | 10184.67 ± 988.14 | | 1439.33 ± 386.33 | | | 23226.33 ± 5526.08 | |
|  | EW3D10 | 1642.67 ± 260.61 | | 938.33 ± 199.42 | | | 2682.333 ± 402.13 | |
|  | EW3F5 | 20009.00 ± 3606.63 | | 2116.00 ± 1019.47 | | | 38707.33 ± 7860.60 | |
|  | EW4E9 | 5282.00 ± 552.39 | | 1221.00 ± 323.24 | | | 11638.67 ± 2126.11 | |
|  | HS3A8 | 7900.67 ± 879.12 | | 1184.33 ± 256.66 | | | 22353.67 ± 3785.48 | |
|  | HS4D4 | 170.33 ± 21.46 | | 514.67 ± 39.26 | | | 221.3333 ± 17.10 | |
|  | HS4E4 | 4795.67 ± 984.66 | | 852.33 ± 122.91 | | | 10837.67 ± 1221.34 | |
|  | LKIV69 | 5670.00 ± 2017.71 | | 1161.67 ± 172.24 | | | 11031.67 ± 3766.66 | |
|  | MPB49 | 191.67 ± 29.96 | | 551.00 ± 65.28 | | | 259.6667 ± 46.36 | |

**Table S1. HS scFv binding intensity in hematopoietic cell lines.** Average mean fluorescence intensity (MFI) of signal along with standard deviation (SD) from each scFv within the 32D, HEL, K-562, MOLM-14, MEL, ES-EP and TF-1 cell lines under basal and differentiation conditions (if applicable) (n=3 for all experiments).
