# Supplementary material for: A glycan-based approach to cell characterization and isolation: Hematopoiesis as a paradigm
Source: J Exp Med. 2022 Sep 6;219(11):e20212552. doi: 10.1084/jem.20212552 (PMC9455685; doi:10.1084/jem.20212552)
Supplement: Table S2 — shows HS scFv binding intensity in murine hematopoietic populations. [file JEM_20212552_TableS2.docx]

|  | **scFv** | Average MFI ± SD | Average MFI ± SD |  |  |
| --- | --- | --- | --- | --- | --- |
|  | AO4B08 | 351.67 ± 59.94 | 412.67 ± 164.66 |  |  |
|  | EW3D10 | 36.57 ± 5.20 | 54.43 ± 13.57 |  |  |
|  | EW3F5 | 440.67 ± 18.48 | 443.33 ± 142.79 |  |  |
|  | EW4E9 | 205.00 ± 6.56 | 328.67 ± 107.04 |  |  |
|  | HS3A8 | 210.00 ± 28.51 | 295.33 ± 116.17 |  |  |
|  | HS4D4 | 25.97 ± 2.21 | 35.57 ± 1.86 |  |  |
|  | HS4E4 | 423.67 ± 33.08 | 435.33 ± 113.50 |  |  |
|  | LKIV69 | 332.00 ± 16.70 | 712.00 ± 178.13 |  |  |
|  | MPB49 | 26.20 ± 1.10 | 34.97 ± 2.15 |  |  |
| HSPC Fractions |  | LSK | CMP | GMP | MEP |
|  | **scFv** | Average MFI ± SD | Average MFI ± SD | Average MFI ± SD | Average MFI ± SD |
|  | AO4B08 | 351.67 ± 59.94 | 412.67 ± 164.66 | 264.67 ± 47.06 | 1058.33 ± 170.03 |
|  | EW3D10 | 36.57 ± 5.20 | 54.43 ± 13.57 | 87.37 ± 23.62 | 78.77 ± 10.29 |
|  | EW3F5 | 440.67 ± 18.48 | 443.33 ± 142.79 | 370.33 ± 71.70 | 1246.00 ± 247.37 |
|  | EW4E9 | 205.00 ± 6.56 | 328.67 ± 107.04 | 229.33 ± 37.54 | 810.33 ± 50.21 |
|  | HS3A8 | 210.00 ± 28.51 | 295.33 ± 116.17 | 187.33 ± 33.65 | 736.33 ± 117.47 |
|  | HS4D4 | 25.97 ± 2.21 | 35.57 ± 1.86 | 71.87 ± 17.36 | 36.43 ± 1.79 |
|  | HS4E4 | 423.67 ± 33.08 | 435.33 ± 113.50 | 294.67 ± 85.44 | 1106.00 ± 266.51 |
|  | LKIV69 | 332.00 ± 16.70 | 712.00 ± 178.13 | 455.33 ± 142.15 | 2239.67 ± 558.03 |
|  | MPB49 | 26.20 ± 1.10 | 34.97 ± 2.15 | 71.97 ± 18.97 | 35.60 ± 1.25 |
| Erythroid Fractions |  | R1 | R2 | R3 | R4 |
|  | **scFv** | Average MFI ± SD | Average MFI ± SD | Average MFI ± SD | Average MFI ± SD |
|  | AO4B08 | 1627.67 ± 158.79 | 1085.33 ± 122.17 | 612.67 ± 50.14 | 434.67 ± 28.73 |
|  | EW3D10 | 85.63 ± 1.29 | 110.00 ± 5.20 | 105.67 ± 7.23 | 97.53 ± 9.10 |
|  | EW3F5 | 2206.33 ± 131.49 | 1262.00 ± 120.40 | 703.33 ± 67.30 | 477.67 ± 8.50 |
|  | EW4E9 | 1113.00 ± 112.77 | 734.00 ± 79.87 | 453.33 ± 45.00 | 320.00 ± 19.29 |
|  | HS3A8 | 1046.67 ± 85.20 | 717.67 ± 62.96 | 422.67 ± 26.54 | 299.67 ± 24.50 |
|  | HS4D4 | 64.67 ± 0.91 | 95.20 ± 6.25 | 98.03 ± 10.36 | 92.37 ± 10.72 |
|  | HS4E4 | 1710.67 ± 65.03 | 1101.67 ± 94.69 | 663.33 ± 34.82 | 469.00 ± 26.15 |
|  | LKIV69 | 3147.67 ± 313.38 | 1690.67 ± 176.54 | 989.33 ± 89.29 | 661.33 ± 41.20 |
|  | MPB49 | 60.73 ± 1.72 | 92.67 ± 5.71 | 97.57 ± 8.45 | 92.03 ± 8.01 |
| Megakaryocyte Fractions |  | CD41+ CD42d- | CD41+ CD42d+ |  |  |
|  | **scFv** | Average MFI ± SD | Average MFI ± SD |  |  |
|  | AO4B08 | 135.00 ± 7.21 | 148.67 ± 10.50 |  |  |
|  | EW3D10 | 51.93 ± 5.52 | 54.90 ± 3.48 |  |  |
|  | EW3F5 | 167.67 ± 4.62 | 194.67 ± 20.21 |  |  |
|  | EW4E9 | 120.33 ± 4.93 | 131.67 ± 3.79 |  |  |
|  | HS3A8 | 117.00 ± 9.64 | 125.67 ± 4.04 |  |  |
|  | HS4D4 | 38.57 ± 1.82 | 35.93 ± 1.11 |  |  |
|  | HS4E4 | 238.00 ± 25.16 | 268.33 ± 17.93 |  |  |
|  | LKIV69 | 305.67 ± 15.95 | 299.00 ± 45.30 |  |  |
|  | MPB49 | 39.80 ± 3.30 | 39.00 ± 1.14 |  |  |

**Table S2. HS scFv binding intensity in murine hematopoietic populations.** Average mean fluorescence intensity (MFI) of signal along with standard deviation (SD) from each scFV within FACS-defined hematopoietic populations (n=3 for all experiments).
