# Supplementary material for: A glycan-based approach to cell characterization and isolation: Hematopoiesis as a paradigm
Source: J Exp Med. 2022 Sep 6;219(11):e20212552. doi: 10.1084/jem.20212552 (PMC9455685; doi:10.1084/jem.20212552)
Supplement: Table S3 — lists primer sequences for mouse gene expression analyses. [file JEM_20212552_TableS3.docx]

| Gene | Forward Primer Sequence | Reverse Primer Sequence |
| --- | --- | --- |
| Mouse |  |  |
| *Fli1* | ATGGACGGGACTATTAAGGAGG | GAAGCAGTCATATCTGCCTTGG |
| *Itgb3* | CCACACGAGGCGTGAACTC | CTTCAGGTTACATCGGGGTGA |
| *Igta2b* | TTCTTGGGTCCTAGTGCTGTT | CGCTTCCATGTTTGTCCTTATGA |
| *Mpl* | AACCCGGTATGTGTGCCAG | AGTTCATGCCTCAGGAAGTCA |
| *Pf4* | ACCATCTCCTCTGGGATCCAT | CCATTCTTCAGGGTGGCTATGAG |
| *Gata-1* | CCCACCTCTATCAGGGCCTA | GAGGTTGTAGGCGATCCCAG |
| *EpoR* | GGGCTCCGAAGAACTTCTGTG | ATGACTTTCGTGACTCACCCT |
| *Ermap* | TGTGGTGGCGTGGAAAATGT | TGTGTCCCTAAGAGGGGCTAT |
| *Klf3* | GAAGCCCAACAAATATGGGGT | GGACGGGAACTTCAGAGAGG |
| *Klf1* | AGACTGTCTTACCCTCCATCAG | GGTCCTCCGATTTCAGACTCAC |
| *Gapdh* | CCAGCCTCGTCCCGTAGAC | GCCTTGACTGTGCCGTTGA |

**Table S3. Primer sequences for mouse gene expression analyses.** Primer sequences used for mouse qRT-PCR experiments.
